# CD Maps—Dynamic Profiling of CD1–CD100 Surface Expression on Human Leukocyte and Lymphocyte Subsets

**DOI:** 10.3389/fimmu.2019.02434

**Published:** 2019-10-23

**Authors:** Tomas Kalina, Karel Fišer, Martin Pérez-Andrés, Daniela Kuzílková, Marta Cuenca, Sophinus J. W. Bartol, Elena Blanco, Pablo Engel, Menno C. van Zelm

**Affiliations:** ^1^CLIP - Childhood Leukaemia Investigation Prague, Department of Paediatric Haematology and Oncology, Charles University, Prague, Czechia; ^2^Department of Paediatric Haematology and Oncology, University Hospital Motol, Prague, Czechia; ^3^Department of Medicine, Cancer Research Centre (IBMCC, USAL-CSIC), Cytometry Service (NUCLEUS), Institute of Biomedical Research of Salamanca, University of Salamanca, Salamanca, Spain; ^4^Biomedical Research Networking Centre Consortium of Oncology (CIBERONC), Instituto de Salud Carlos III, Madrid, Spain; ^5^Department of Biomedical Sciences, University of Barcelona, Barcelona, Spain; ^6^Department of Immunology, Erasmus MC, University Medical Center, Rotterdam, Netherlands; ^7^Department of Immunology and Pathology, Monash University and the Alfred Hospital, Melbourne, VIC, Australia

**Keywords:** CD marker, surfaceome, lymphocyte, monocyte, flow cytometry, expression profiling, B-cell, T-cell

## Abstract

CD molecules are surface molecules expressed on cells of the immune system that play key roles in immune cell-cell communication and sensing the microenvironment. These molecules are essential markers for the identification and isolation of leukocytes and lymphocyte subsets. Here, we present the results of the first phase of the CD Maps study, mapping the expression of CD1–CD100 (*n* = 110) on 47 immune cell subsets from blood, thymus, and tonsil using an eight-color standardized EuroFlow approach and quantification of expression. The resulting dataset included median antibody binding capacities (ABCs) and percentage of positivity for all markers on all subsets and was developed into an interactive CD Maps web resource. Using the resource, we examined differentially expressed proteins between granulocyte, monocyte, and dendritic cell subsets, and profiled dynamic expression of markers during thymocyte differentiation, T-cell maturation, and between functionally distinct B-cell subset clusters. The CD Maps resource will serve as a benchmark of antibody reactivities ensuring improved reproducibility of flow cytometry-based research. Moreover, it will provide a full picture of the surfaceome of human immune cells and serves as a useful platform to increase our understanding of leukocyte biology, as well as to facilitate the identification of new biomarkers and therapeutic targets of immunological and hematological diseases.

## Introduction

Leukocytes display on their surface molecules that are crucial for sensing hazardous environmental changes and mediating cell adhesion and communication between cells both within the immune system and with stroma. These include receptors, transporters, channels, cell-adhesion proteins, and enzymes. The complexity of surface-expressed proteins, also called the surfaceome, is emphasized by the fact that an estimated 26% of human genes encode transmembrane proteins (~5,500) (1). However, recent *in silico* evaluations predict that 2,886 proteins are actually expressed at the outer cell membrane, i.e., the cell surface (2). Experimental evidence exists for ~1,492 proteins across multiple tissues (3) and 1,015 proteins that are expressed in one or more immune cell type and lymphoid tissue (4).

Over the past four decades, a vast array of cell surface molecules has been discovered through the production of monoclonal antibodies (mAbs) (5). These mAbs, together with the development of multicolor flow cytometric analysis (6), have been instrumental to determine their expression and function. Human leukocyte differentiation antigen (HLDA) workshops have led to the characterization and formal designation of more than 400 surface molecules (7, 8), known as CD molecules (www.hcdm.org). CD nomenclature provides a unified designation system for mAbs, as well as for the cell surface molecules that they recognize. These molecules include receptors, adhesion molecules, membrane-bound enzymes, and glycans that play multiple roles in leukocyte development, activation, and differentiation. CD molecules are routinely used as cell markers, allowing the identification of the presence and proportions of specific leukocyte cell populations and lymphocyte subsets, and their isolation, using combinations of fluorochrome-labeled antibodies and flow cytometry. Importantly, analysis of CD molecules, known as immunophenotyping, is a fundamental component for the diagnosis, classification, and follow-up of hematological malignancies and immunodeficiencies, and the monitoring of immune system disorders such as autoimmune diseases. More recently, mAbs recognizing CD molecules have been established as invaluable tools for the treatment of cancer, such as checkpoint inhibitors (9), and autoimmune diseases (10). Development and testing of such therapeutics rely on accurate knowledge expression and function of the target molecule as has been negatively illustrated by the disaster in the Phase I TGN1412 study with an anti-CD28 superagonist (11).

Currently, there are extensive gaps in our knowledge of CD molecule expression patterns, mainly because of the discordancy in the setup of the expression studies and the major changes in flow cytometry technology over the last 30 years (12). As a result, there has been overinterpretation in summarizing tables, which can be misleading. Thus, there is an urgent need to construct a higher resolution and accurate map of the expression profiles of the CD molecules to visualize the surface of leukocyte landscape. Moreover, an important part of the bibliography is incorrect and often misleading.

To correct current misinterpretation and to overcome gaps in knowledge, the HCDM has initiated the CD Maps project, a multi-institute research program to generate a high-resolution map of the cell surface of human immune cells using standardized multicolor flow cytometry protocols. Here, we present the results of the first phase of the CD Maps study, which includes the expression signature of CD1–CD100 on 47 cell populations and subsets, 41 of which were non-overlapping. The data have been acquired across four expert flow cytometry laboratories to ensure reproducibility and have been built into an online web resource with free user access. Expression profiling of CD markers across immune cell subsets revealed dynamic changes in expression levels and hints at further immune cell diversity for markers that were expressed on a fraction of defined populations. These insights can prove critical for development of therapeutics targeting dysregulated immune responses or malignant cells.

## Materials and Methods

### Human Tissue Samples

The use of human pediatric tissue and adult buffy coats was approved by the Human Ethics Committees of the Erasmus Medical Center, the University Hospital Motol, and the universities of Salamanca and Barcelona, and was contingent on informed consent in accordance with the Declaration of Helsinki. Thymus material was obtained from 12 children requiring surgery for congenital heart disease. These children did not have hematologic or immunologic diseases. Non-necrotizing tonsil tissue was obtained from seven donors, including two adults (32 and 34 years) and five children (4–8 years) who underwent scheduled tonsillectomy. Blood buffy coats of 12 healthy adult volunteer donors were obtained from the local blood banks.

### Single Cell Isolation and Preparation

The blood leukocyte isolation protocol was optimized to minimize platelet adhesion (satellitism). Briefly, the buffy coat suspension was diluted 6 × in PBS containing 2 mM EDTA, followed by adding an equal volume of a 4% dextran solution (Sigma-Aldrich, Saint Louis, MO, USA) in 0.9% NaCl. The mixture was left for 30 min for erythrocytes to sediment prior to collecting the supernatant containing the leukocytes. Following a spin (130 *g*, 15 min, RT) and removal of the supernatant, the residual erythrocytes in the pellet were lysed using hypotonic lysis with a 0.2% NaCl solution for 55 s, followed by supplementation of 1.2% NaCl to achieve an isotonic concentration of NaCl. Following addition of PBS and a spin (130 *g*, 15 min, RT), the lysis step was repeated. Finally, the suspension of leukocytes was washed and diluted with PBS/BSA (PBS with 0.5% BSA and 0.09% NaN_3_) to a final concentration of 4 × 10^7^/ml.

Thymocytes and tonsillar lymphocytes were isolated via gentle shaking from manually dissociated thymus and tonsil tissue, respectively, washed with RPMI 1640 with 25 mM HEPES, L-glutamine, 100 U/ml penicillin, and 100 mg/ml streptomycin (Lonza, Basel, Switzerland) supplemented with 10% (v/v) heat-inactivated fetal bovine serum (FBS, Thermo Fisher Scientific, Rockford, IL). Single-cell suspensions were either directly used for immunophenotyping, or stored in FBS with 10% DMSO in liquid nitrogen for analysis at a later stage. Live frozen cells were thawed by dropwise addition of 1 ml FBS, followed by addition of 8 ml of medium. Cells were washed twice, counted, washed, and diluted with PBS/BSA (PBS with 0.5% BSA and 0.09% NaN_3_) to a final concentration of 1.25 × 10^7^/ml. Whenever frozen and thawed thymocytes were used, we observed a marked decrease of proportion of double-positive stage thymocytes, but their phenotype was similar to the fresh thymocytes.

### Staining of Cells With Antibodies for Immunophenotyping

Cells were stained in V-bottom 96-well plates in a total suspension volume of 50 μl. First, one of each of the PE-labeled mAbs to CD1–CD100 were added to each well (details of each marker are provided in Supplementary Table 2). The amounts were according to the manufacturer's recommended titer and topped up to 10 μl with PBS/BSA. Subsequently, 40 μl of cell suspension (1.6 × 10^6^ cells for buffy coats, 5 × 10^5^ cells for thymus or tonsil) was added to each well. Following careful mixing, the suspensions were incubated for 30 min at room temperature in the dark. Next, 25 μl of backbone mAb reagent mix was added to each well, carefully mixed, and incubated again for 30 min (RT, in the dark). Four Ab backbone cocktails were prepared (two for blood, one for thymus, and one for tonsil), and the reagents were titrated beforehand (details provided in Supplementary Table 1). The cells were washed three times (8 min, 500 *g*, RT) in PBS/BSA and resuspended in 200 μl of PBS with 2 mM EDTA for acquisition. A detailed CD Maps standard operating protocol can be downloaded from www.hcdm.org. Although we aimed for the complete set of CD1–CD100 markers, we were limited to the 110 that were commercially available and that were not of the IgM isotype. The following CD markers were not included: (a) mAbs with IgM isotype against carbohydrate antigens that were not available as PE-conjugates: CDw12, CD15u, CD15s, CD15su, CD17, CD60a, CD60b, CD60c, CD65, CD65s, CD75, and CD75s; (b) mAbs that were validated by the HLDA workshops, but that were not commercially obtainable: CD1c, CD66a, CD66d, CD66e, CD66f, CD85a, CD92, and CD94. Furthermore, several CD markers were present as backbone markers in our panels potentially interfering with the PE staining. To mitigate the blocking effect on the PE-reagent, we (a) used a different clone known to bind a distinct epitope (e.g., CD16, CD45), and where no clone with a distinct epitope was available, we (b) incubated the cells first with the PE-conjugate for 15 min, prior to addition of the backbone cocktail. When the backbone marker was impacted, the gating strategy was manually adjusted using the PE-conjugated marker. The CD1–CD100 markers were assessed with commercially available reagents from three different vendors and used at vendor-recommended titers. Some reagents exhibited higher background staining than others, which is probably due to these having a lower antigen affinity and were therefore used at higher concentration. This could explain why the expression levels (MFI) for some CD markers were above that of the FMO in a subset that is known not to express it. Finally, some subsets (particularly myeloid cells and cells from tonsil) exhibited high background autofluorescence and some degree of non-specific binding (13).

### Flow Cytometer Instrument Setup

Data acquisition was performed on four different sites on LSR II, LSR Fortessa, and FACS Canto instruments (BD Biosciences, San Jose, CA, USA) equipped with 405-nm, 488-nm, and 633/647-nm excitation lasers and an HTS loader. Cytometer Setup and Tracking (CS&T) beads (BD Biosciences) and 8-peak Rainbow bead calibration particles (Spherotech, Lake Forest, IL, USA) were used for PMT voltages and light scatter setup to achieve inter-laboratory standardization as developed by the EuroFlow consortium (14). Each panel was applied on a total of 12 donors, and 1 million events were acquired per staining (well). The EuroFlow Standard Operating Procedure (SOP) for Instrument Setup and Compensation can be downloaded from www.euroflow.org. Three out of four laboratories participate in the EuroFlow Quality Assessment scheme that investigates the MFI of selected cell subsets (15). The same concept was adopted to test the performance of the four laboratories on a testing cohort of three local donors using four reagents (CD8, CD21, CD25, and CD28) representing different staining intensities.

### Conversion of PE Fluorescence Intensity to Antibody Binding Capacity (ABC)

To convert PE fluorescence to the amount of PE molecules bound to a target, we used the PE Fluorescence Quantitation Kit (BD Biosciences) with four known levels of PE. The pellet was resuspended in 500 μl of PBS/BSA and analyzed by flow cytometry in parallel with each experiment. The measured PE signals for all stainings on all cell subsets were fitted to the PE calibration curve to extract the number of PE molecules.

PE-conjugation of mAbs is quite consistent with a 1:1 ratio of fluorochrome:antibody. To test and correct for any deviations, we have measured and calculated a correction factor reflecting the amount of PE for each antibody (correction factors were in the range 0.73–1.32, mean – 1 SD to mean + 1 SD). A volume of 25 μl of UltraComp eBeads™ Compensation Beads (Thermo Fischer Scientific) was diluted with 15 μl of PBS/BSA, mixed with excess of tested PE-labeled antibody and incubated for 30 min, RT, in the dark. Compensation Beads were washed twice in PBS/BSA (8 min, 500 *g*, RT), resuspended in 70 μl of PBS with 2 mM EDTA, and analyzed by flow cytometry. All 116 mAbs were measured, and for each mAb, a ratio of individual median PE/(median of all medians) was calculated as a correction factor. A standard deviation of the correction factor was 0.3; a total of 26 mAbs (25%) of all mAbs yielded a correction factor above or below 1 standard deviation; thus, for mAbs with a correction factor <0.7 or above 1.3, the measurement was repeated to exclude any outliers. The average of all correction factor values (after exclusion of outliers) was used to recalculate the ABC for all 111 CD markers on all 47 defined subsets.

### Analysis, Gating, and Export of Values

Leukocyte and lymphocyte subsets to be analyzed were pre-defined (Supplementary Figures 1–4), and all acquisitions for each of the four panels were gated by a single laboratory using FlowJo (version 9 or 10) or Infinicyt software. From each defined subset, the following set of statistics was extracted for the PE channel: median, mean, mode, CV, 10th, 25th, 50th (median), 75th, 90th percentile (Supplementary Figure 5). Furthermore, a gate was set to define the percentage of positive events, using the fluorescence minus one (FMO) staining as a negative control. The minimum cell count for statistical evaluation was set to 100, and subsets with lower cell counts were omitted from further analysis. Samples with <500,000 events in the leukocyte gate (CD45+) or samples with an apparent shift in CD45 expression with time during acquisition (indicative of clogging) were not used for analysis (manually curated).

The conversion from PE fluorescence to target molecule number (ABC unit) was performed as described above using the “define calibration” function in either the FlowJo or Infinicyt software packages. Descriptive statistics obtained from these software programs were exported for all defined subsets into one delimited flat table text file per tube. To these tables, additional information on material source, antibody characteristics, experiment details, etc. were added, as well as uniform cell subset identifiers: short machine friendly names, longer descriptive names.

### Data Import and Pre-processing

All subsequent work was carried out in R Development Core Team (16). All used R packages are listed and references are provided in Supplementary Table 4. Data were imported into the R environment using standard import functions, converting data to R objects. Each of the four data flat tables from the four tubes was processed separately. After checks for duplicated data entries, these were converted into matrix-like formats and previously calculated median correction factors were applied. Sample wise centrality measures (means and medians) were calculated and data were converted from wide to long format for easier subsequent computation. Dictionaries of cell subset and statistics-related terms were built and combined from all sources. The processed and combined data were stored in binary format and were cleaned (all non-positive values were converted to the value one), a correction factor was applied, and group-based centrality statistics (mean and median) were calculated.

### Distribution of Frequency of PE-Positive Cells

Sigmoidal fit and separation of markers into positive, intermediate, and negative groups on a per-cell subset basis was performed using R package sicegar (17). Simple sigmoidal fit was performed by logistic function

$$\begin{array}{l} PE\left(cds\right)=f_{sig}\left(cds\right)=\frac{PE_{\max}}{1+\;\exp\left({-a}_{1}\left(cds-cds_{mid}\right)\right)} \end{array}$$

where *PE(cds)* is the percentage of PE-positive cells, given as a function of sequence of CD markers *cds*. The CD markers are ordered based on rising median percentage of PE-positive cells. There are three parameters to be fitted: *PE*_*max*_—maximum percentage of PE-positive cells, *cds*_*mid*_—midpoint as half of maximum, and *a*_1_. The *a*_1_ parameter is related to the slope of *PE(cds)* at *cds* = *cds*_*mid*_ via the formula.

$$\begin{array}{l} \frac{d}{dcds}PE\left(cds\right){|}_{{cds=cds}_{mid}}=\frac{a_{1}PE_{\max}}{4} \end{array}$$

### Distribution of Median Fluorescence Intensity

Modeling of a turning point in a sequence of rising median fluorescence intensity per cell subset was done using Menger curvature adapted from Christopoulos (18).

The Menger curvature for *y* = *f*(*x*) at (*x*_*i*_, *y*_*i*_) is:

$$\begin{array}{l} DC\left(x_{i}\right)=\frac{\sqrt{A-B^{2}}}{\left\|pq\|\|qr\|\|rq\right\|} \end{array}$$

where

$$\begin{array}{l} A=4{\left\|pq\right\|}^{2}{\left\|qr\right\|}^{2}\\ B={\left\|pq\right\|}^{2}+{\left\|qr\right\|}^{2}-{\left\|rp\right\|}^{2}\\ \|pq\|=\sqrt{{\left(x_{i-1}-x_{i}\right)}^{2}+{\left(y_{i-1}-y_{i}\right)}^{2}}\\ \|qr\|=\sqrt{{\left(x_{i}-x_{i+1}\right)}^{2}+{\left(y_{i}-y_{i+1}\right)}^{2}}\\ \|rp\|=\sqrt{{\left(x_{i+1}-x_{i-1}\right)}^{2}+{\left(y_{i+1}-y_{i-1}\right)}^{2}} \end{array}$$

And the convex turning point at section of the curve is:

$$\begin{array}{l} D=max\left\{DC\left(x_{i}\right),i=2,\ldots ,n-1\right\} \end{array}$$

### Hierarchical Clustering Analysis

For hierarchical clustering analysis (HCA), the pheatmap R package (https://github.com/raivokolde/pheatmap) was used. Per cell subset, median Qb values were log_10_ transformed after minimum median Qb values were raised above zero. Observations with missing values and FMO controls were removed and data were *z*-score scaled. For HCA, the Euclidean distance and Ward linkage (ward.D2) were used (19).

### Generation and Utilities of a Dynamic Web Resource

**Figure 1:**
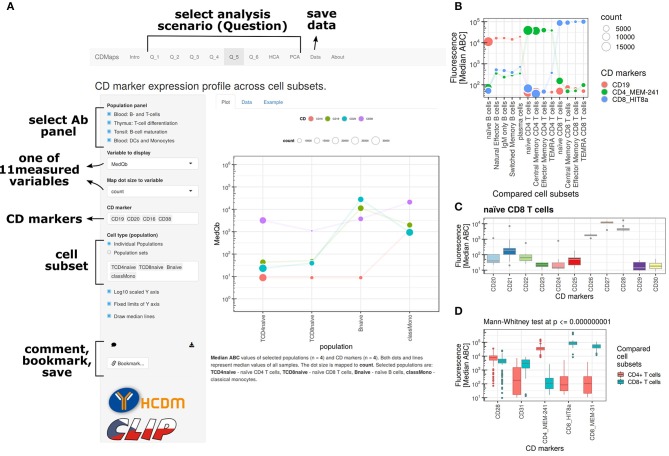
Overview of the CD Maps web resource available at hcdm.org. **(A)** The user interface allows for interactive data interrogations. The resource uses several scenarios for data exploration and allows for data download, bookmarking of analysis state, and image export. Analysis examples: **(B)** paired analysis of CD marker expression on cell subsets, e.g., in a differentiation setting. **(C)** Visualization of expression of multiple CD markers including a measure of variation for a single subset. **(D)** Analysis and visualization of statistically differentially expressed CD markers between two subsets or two groups of cell subsets.

To share CD Maps data as a resource with a user-friendly interface, an application with web page front-end was written in R using the R package Shiny. Shiny allows background computations in R serving results to a web-based front-end and uses a reactive programming paradigm. Reactive programming allows for dynamic user-directed content generation and therefore interactive data exploration and analysis. For enhanced user interactivity, several R packages were used that facilitate access to JavaScript libraries (e.g., d3heatmap, htmlwidgets). The resulting web page includes general CD Maps information, as well as several angles from which to interrogate CD Maps data (www.hcdm.org; Figure 1).

An example is the interrogation feature “What are protein levels of selected CD markers on selected sequence of cell subsets?” For this scenario, the user is able to select CD markers and a sequence of cell subsets to visualize expression in multiple subsets using a dot-line plot. The sequence of cell subsets is based on the order in which these have been selected, and the values on the *y* axis are by default the median ABC values from all biological repeats. The variable displayed on the *y* axis can be exchanged by the user for any of the available cell subset statistics. As the graph is also a dot plot, the size of the dots can be used to visualize an additional quantitative parameter per cell subset and can be selected by the user (e.g., percentage of PE-positive cells). The line plot uses unique colors for each selected CD marker. Besides the graph itself, the application also dynamically generates figure captions. Finally, the application also allows the user to “bookmark” the state selected settings in the application for later follow-up analysis. In conclusion, the web resource functions are based on the principle that the user specifies details for data interrogation within given scenario boundaries, and such details are sent to the web server, where R is used to compute and prepare outputs, and those outputs are sent back in real time to user, giving a smooth, dynamic, and interactive feeling to the user.

### Reproducibility and Version Control

Reproducibility and version control of data processing and application development throughout the project were achieved using GIT versioning software (https://git-scm.com/) RStudio IDE (RStudio, Inc., Boston, MA, USA) and Bitbucket repository (Atlassian, Sydney, Australia). Deployment is facilitated via Docker virtualization (https://www.docker.com/, Docker, Inc., San Francisco, CA, USA).

## Results

### Generation of a Web Resource for Expression Profiling of CD1–CD100 on Major Immune Cell Lineages and Their Subsets

To investigate the expression levels on major leukocytes, subsets of the first surface molecules that had been defined in the 1980s and early 1990s with CD markers 1–100 (20–24), we developed a multicolor immune phenotyping panel consisting of four tubes: (A) innate and (B) adaptive immune cells from blood (25, 26), (C) B-cell subsets from tonsil (27, 28), and (D) T-cell progenitors in thymus (29, 30) (Supplementary Table 1). One channel was reserved for a PE-labeled drop-in mAb directed against one of the CD1–CD100 antigens (Supplementary Table 2). Twelve biological repeats were acquired, and after curation (detailed SOP in Supplementary Data Sheet 1), expression analysis was performed on nine biological repeats for tube A, 11 for B, 7 for C, and 5 for D. Multiple descriptors of CD marker expression were defined for each gated cell subsets and exported (Supplementary Figure 5), including the median fluorescence intensity, which was converted to ABC using the QuantiBRITE bead measurements, and the percentage of positive cells using the FMO control value as cutoff.

The resulting dataset consisted of over a million data points of derived statistics and annotation information that together form a quantitative insight into the cell surfaceome of the human immune system. To make the data accessible as a major resource for detailed studies by us and the scientific community, we constructed an interactive web-based application (Figure 1). The resource contains multiple features to visualize the complete dataset [e.g., principal component analysis (PCA)] and to examine specific cell lineages and/or subsets (e.g., pairwise comparisons and patterns of expression during cell maturation).

**Figure 2:**
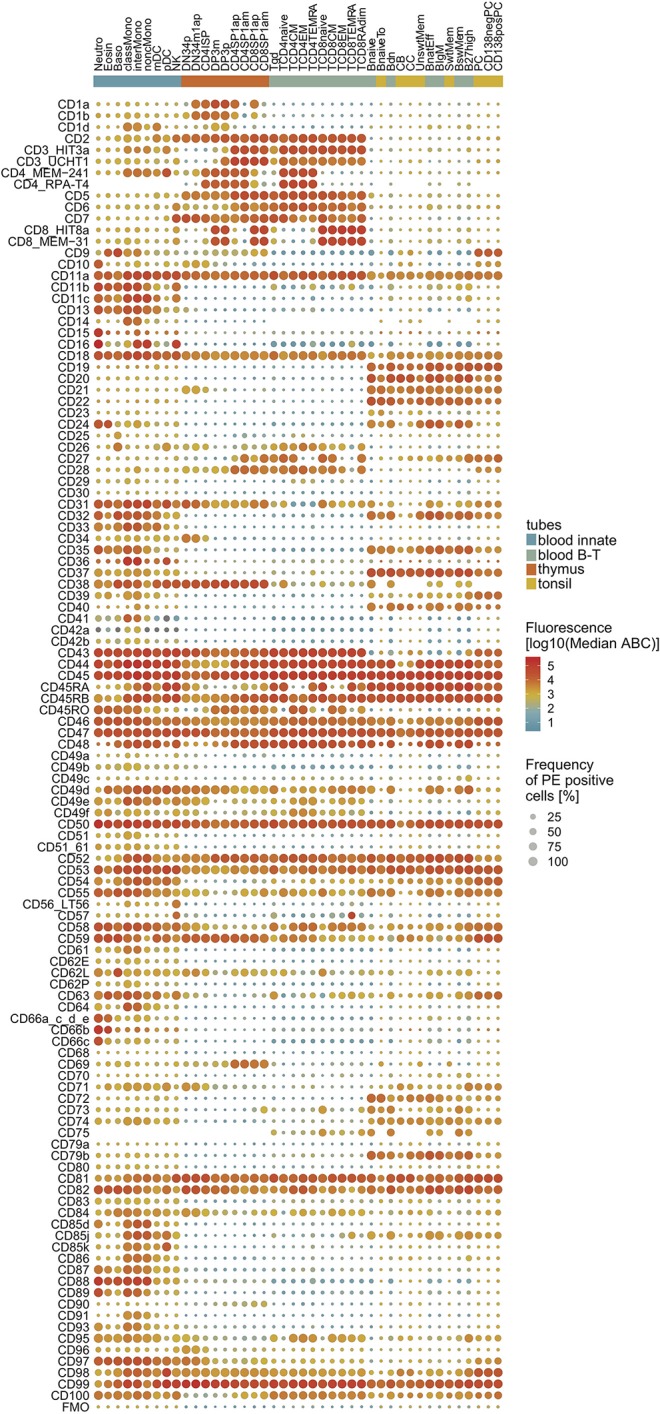
Expression map of CD1–CD100 on all 42 non-overlapping cell subsets. CD markers are numerically ordered vertically with the FMO on the bottom row. The cell subsets are grouped (innate cells; thymocytes; T-cells; B cells) and sorted within lineage on their maturity. The median expression level is visualized by color, and the median percentage of positive cells is visualized by the size of the dot. For cell type abbreviations, see Supplementary Table 3.

The combined information of CD marker expression levels and percentages of positive cells were depicted as a “drop plot” (Figure 2), in which colors represent the ABC and the dot sizes represent the percentage of positivity. The CD markers displayed a wide range of expression patterns. For example, CD44, CD45, CD46, and CD47 were highly expressed on nearly all cells within the majority of defined subsets, whereas CD49a, CD49b, and CD49c were typically expressed at low levels. Importantly, all markers showed positivity for at least one subset, and the expression patterns of molecules such as CD3, CD4, CD8, CD14, CD19, and CD20 agreed with their designation as well-defined lineage markers (Figure 2).

### Intra- and Inter-population Variation of CD Marker Expression

**Figure 3:**
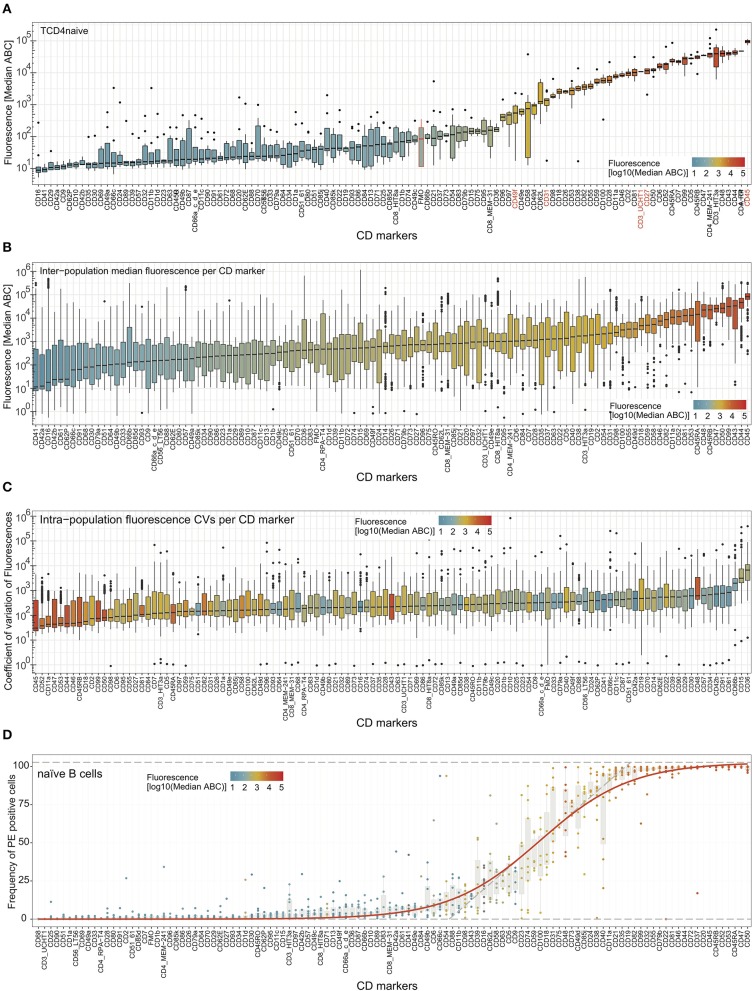
Expression levels and heterogeneity of expression of cell surface markers across cell types. **(A)** Median fluorescence (in antibody binding capacity; ABC) for all markers on one cell subset (naïve CD4 T-cells) ordered from low to high median expression. CD markers in red font are discussed in the main text, FMO is highlighted in orange, and a horizontal orange line depicts the median FMO background. Similar plots for all cell subsets are provided in Supplementary Figure 6. **(B)** Fluorescence (in ABC) across all cell subsets per CD marker with box whisker plots (median, IQR, and range). The CD markers are ordered from low to high median expression (black horizontal lines). **(C)** Coefficients of variation (CV) of expression across all cell subsets per CD marker. The CD markers are ordered from low to high median CV (black horizontal lines) as box whisker plots with the color representing the median expression level ABC. **(D)** Frequency of positive cells for all markers on one cell subset (naive B-cells) ordered from low to high frequency. Similar plots for all cell subsets are provided in Supplementary Figure 7. In all plots, fluorescence intensity is also represented by the coloring of the boxes.

Further examination involved the relative intensity of expression of all CD markers in all defined cell subsets (Supplementary Figure 6 and Supplementary Table 4). The most highly expressed markers (e.g., CD45 on naive CD4 T-cells; Figure 3A) reached 10^5^ ABC units, with lower expression levels for, e.g., CD3 and CD27 at 10^4^, and CD31 and CD49f at 10^3^. Ubiquitously expressed molecules on immune cells such as CD44, CD45, CD46, CD47, CD50, CD98, and CD99 had a low coefficient of variation (CV) across the studied subsets (Figure 3B), as did some molecules with overall low expression levels (e.g., CD49c). In contrast, as expected, markers with lineage- and/or subset-specific expression patterns show a greater degree of heterogeneity in expression over the examined subsets (e.g., CD19, CD24, CD35).

To examine donor variation for expression all markers, CVs were calculated per cell subset for each marker and displayed as box whisker plots (Figure 3C). In general, the highly expressed markers were found to have relatively low inter-donor variability, whereas the CVs were higher for CD markers that were expressed at low levels (Figure 3C). Indeed, some of the markers with small boxes in Figure 3A (CD44, CD45, CD46, CD47, CD98, and CD99) were highly expressed and showed a relatively low CV. Still, some CD markers had a higher variability of expression in all cell subsets (CD15, CD36, and CD66b), and some CD markers with higher ABC had also relatively high CVs (CD43 and CD48).

The amount of surface protein (here expressed as ABC) is perhaps the most used measure of protein expression in a cell subset and corresponds most closely to measures of expression in other forms of analysis with bulk cells. However, flow cytometry being a single-cell technique has the advantage of distinguishing individual cells that do or do not express a marker. This can be shown as percentage of positivity, and this has been defined relative to FMO for all measured CD markers in each cell subset (Supplementary Figure 7 and Supplementary Table 5). Ordered visualization of markers with increased positivity revealed sigmoidal curves per cell subset (Figure 3D and Supplementary Figure 7), separating markers that were negative on all, positive on all, or positive on a fraction of the cells within the subset. The frequencies of positive cells were tightly associated with the fluorescence (shown by coloring), with some exceptions: e.g., low CD9 and high CD48 on naive B-cells (Figure 3D).

### Clustering of Cell Subsets and CD Markers

**Figure 4:**
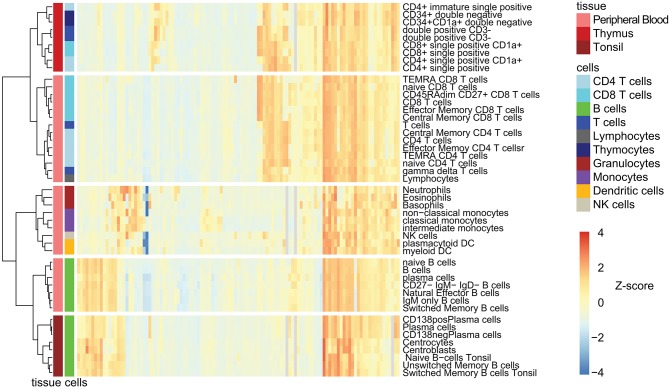
Hierarchical clustering analysis of CD marker expression on leukocyte subsets. Unsupervised clustering was performed on all CD markers (*n* = 117) and all cell subsets (*n* = 47) based on log_10_ transformed Median ABC. Cell subsets are color coded based on their lineage and their tissue of origin. Hierarchical tree was algorithmically cut into clusters (*k* = 5). Each cell subset group could contain cell subset from one or more tissue type (peripheral blood, thymus, and tonsil). See Supplementary Figure 8 for larger version of this figure, including CD marker labeling.

To interrogate and visualize common expression patterns of markers and how these related on the defined cell subsets, we performed unsupervised HCA (Figure 4). The analysis revealed three main cell clusters: T-cells, B-cells, and myeloid cells. Within both B- and T-cells, the blood and tissue subsets were grouped into two separate subclusters.

Regarding CD marker patterns, CD19, CD20, CD21, CD22, CD72, and CD74 clustered together with predominant expression among B-cell subsets, whereas CD11b, CD11c, CD13, CD14, CD16, CD33, and CD88 were found to be expressed in the myeloid cell cluster (Supplementary Figure 8). The thymocyte cluster contained CD9, CD10, CD1a, CD1b, CD1d, CD71, CD69, CD90, and CD34, which are known markers for progenitor cells and for cell activation. A cluster of CD markers expressed on all subsets and at all stages included CD45, CD44, CD99, CD47, and CD50. Lastly, a T-cell cluster was apparent, containing CD2, CD3, CD4, CD5, CD6, CD7, CD8, CD26, CD28, CD49e, CD49f, CD62L, CD84, CD95, and CD96. In addition to these dominant clusters, the heatmap also clearly visualizes expression of CD markers outside of the dominant cluster, such as CD24 expression on neutrophils and eosinophils, and CD21 expression on immature thymocytes (Figure 4 and Supplementary Figure 8).

### Granulocyte, Monocyte, and Dendritic Cell Analysis

**Figure 5:**
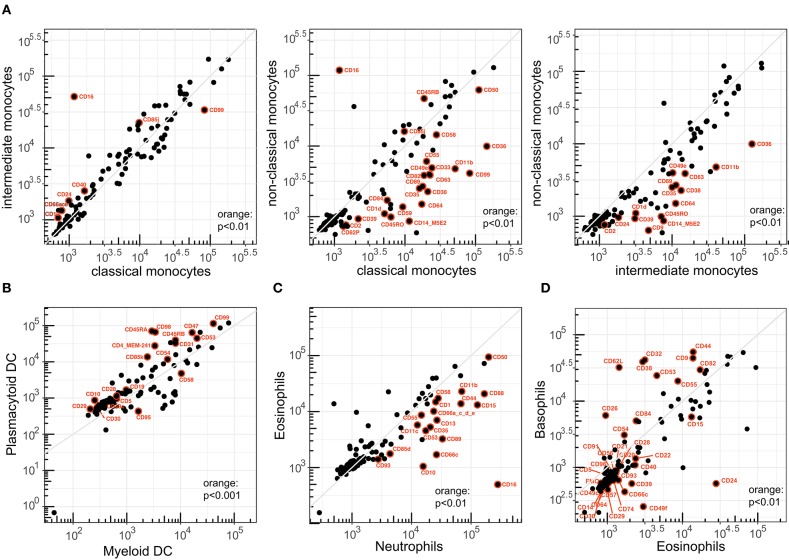
Correlation of CD1–CD100 expression levels between related innate leukocytes. Pairwise analysis of expression levels (ABC) between **(A)** classical, intermediate, and non-classical monocytes; **(B)** myeloid DC and plasmacytoid DC; **(C)** neutrophils and eosinophils; and **(D)** eosinophils vs. basophils. CD41 and CD42b were excluded from the plots in **(B–D)**. Both markers were not expressed on any granulocyte or DC cell type (ABC < 2 × 10^2^).

Three monocyte subsets can be typically defined based on differential expression of CD14 and CD16 (Supplementary Figure 1), and these subsets have been shown to be associated with distinct diseases (31, 32). Of the 111 CD markers tested, 31 were significantly different in ABC (*p* < 0.01) between any two of the three subsets (Figure 5A). Remarkably, multiple integrins (CD11b, CD49e) and other adhesion molecules (CD33, CD62P), as well as antigen-presentation molecule CD1d were specifically downregulated on non-classical monocytes as compared to the classical and intermediate subsets.

By definition, CD16 (FcγRIII) was upregulated on intermediate and non-classical monocytes. In contrast, CD64 (FcγRI) was specifically downregulated on non-classical monocytes, whereas all subsets expressed relatively similar levels of CD32 (FcγRIIa and FcγRIIb). The CD35 antigen (complement receptor 1) was specifically downregulated on non-classical monocytes. Within the family of tetraspanins, CD63 expression was specifically high on classical monocytes, and CD9 and CD82 expression levels were significantly reduced on non-classical monocytes, whereas no differences were seen for CD37, CD53, and CD81.

Similar to the monocyte subsets, we performed a detailed phenotypic comparison between the major two DC subsets in blood: myeloid (m)DC and plasmacytoid (p)DC. pDCs were defined on the basis of co-expression of HLA-DR and CD123 (Supplementary Figure 1 and Supplementary Table 3). Due to the limitations in markers we could use in the backbone, we defined one mDC population on the basis of HLA-DR+CD11c+CD14–CD16–, which includes both the CD1c+ cDC1 and the CD141+ cDC2 subsets (33). Forty of the 111 CD molecules differed significantly in expression level between mDC and pDC (*p* < 0.01), and of these 19 with a *p* < 0.001 (Figure 5B). Most of the differences were the result of higher expression of markers on pDCs. Markers with low expression included molecules typically found on lymphocytes (CD3, CD10, and CD19), and this probably does not represent actual expression. In addition, pDC expressed higher levels of multiple integrins (CD29, CD49a, CD49c, CD49d) and adhesin molecule CD54 (ICAM-1), as well as the previously reported immunoregulatory receptor CD5 and tolerogenic receptors CD85d, CD85j, and CD85k (33), whereas the death receptor CD95 was significantly reduced on pDC (34). Expression levels of the previously reported CD11b, CD11c, and CD13 were reduced, but not with a significance of *p* < 0.01 (34).

Between neutrophils and eosinophils, 20 CD molecules were significantly different (*p* < 0.01) and all were lower on the latter subset (Figure 5C). These included the well-described CD10, CD15, and CD16, as well as integrins CD11b, CD11c, CD18; integrin ligand CD50; complement receptors CD35, CD88, and CD93; and the IgA receptor CD89. About half of the significantly different markers between basophils and eosinophils were around borderline expression (10^3^) (Figure 5D). Of the rest, 11 were significantly higher in basophils and included the tetraspanins CD9, CD53, and CD82; the FcγRII (CD32); multiple cell adhesion molecules (CD38, CD44, CD54, CD62L); complement decay factor CD55; and SLAM family member CD84. Conversely, eosinophils expressed significantly more CD15, glycoproteins CD22 and CD24, ectoenzyme CD39, TNF receptor CD40, and adhesion molecules CD49f and CD66c.

### T-Cell Maturation

Within the CD3+ cells, the three main lineages (TCRγδ+, CD4+, and CD8+) were distinguished (Supplementary Figure 2). Pairwise analysis of parallel maturation stages between the CD4 and CD8 lineages for markers with significance of >0.01 and change of at least 10-fold (Supplementary Figure 9A) revealed consistently higher CD59 expression on CD4 T-cells (all stages, except for TemRA; CD45RA+CD27–) (35). Conversely, “senescence” marker CD57 and tetraspanin CD63 were both higher on CD8 T-cells in the central memory (Tcm) stage.

**Figure 6:**
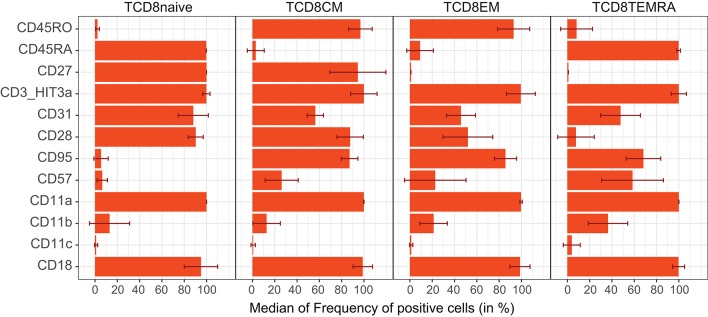
Expression patterns of selected CD markers on naïve and memory CD8 T-cells. Selected CD molecules are shown for naive, central memory (CM), effector memory (EM), and CD45RA+ effector memory (TEMRA) CD8 T-cells. In contrast to co-stimulatory molecules (CD27, CD28) and FasR (CD95), the integrins (CD11a, CD11b, CD11c, CD18) are expressed on all stages of CD8 T-cells; however, their expression levels differ (see Supplementary Figure 8).

In addition, multiple CD markers were differentially expressed between stages of T-cell maturation. Naive CD8 T-cells (CD45RA+CD27+) were nearly all positive for the CD45RA isoform, CD31 (PECAM-1), and costimulatory molecules CD27 and CD28 (Figure 6) (36). While the integrins (CD18 and CD11c) were expressed on all T-cell subsets, their degree of expression increased with maturation (Supplementary Figure 9B). The relative amount of surface CD45RA was about twice as high as CD3, which in turn was nearly twice that of CD27 (Supplementary Figure 9B). The expression levels of regulators of activation were tightly controlled as evidenced by low CV within each subset (CD3, CD45RA, CD28, CD27, and CD31; Supplementary Figure 9C). By definition, CD8 Tcm and Tem cells lacked surface CD45RA, and all expressed the CD45RO isoform, generated by alternative splicing. CD95 was expressed on all memory subsets, whereas CD57 was gradually upregulated from Tcm to Tem subsets, which in turn gradually lost CD31. Furthermore, CD28 positivity decreased from Tcm to Tem. Finally, in TemRA, CD45RA was re-expressed with a concomitant loss of CD45RO, and a massive increase in CD57 positivity (37). In our gating strategy, a separate population (CD45RA^dim^CD27+) was defined in-between CD8 Tnaive and Tcm. In contrast to Tnaive, CD45RA^dim^ cells expressed CD95 and CD45RO and lower levels of CD27, and lacked CD38 expression. On the other hand, the CD45RA^dim^ cells were distinct from TemRA, as they did express CD28, and not CD85j. The phenotype of CD45RA^dim^ cells therefore seems to fit with that of antigen experienced T memory stem cell subset as has been suggested before (38, 39). Similar to CD8 T-cells, transition of naive CD4 T-cells to memory was accompanied by a decrease in expression of CD31, CD38, and CD45RA, while CD45RO, CD95 (Fas-receptor), and CD84 (SLAMF5) were upregulated (data not shown) (40, 41).

### Thymocyte Differentiation

**Figure 7:**
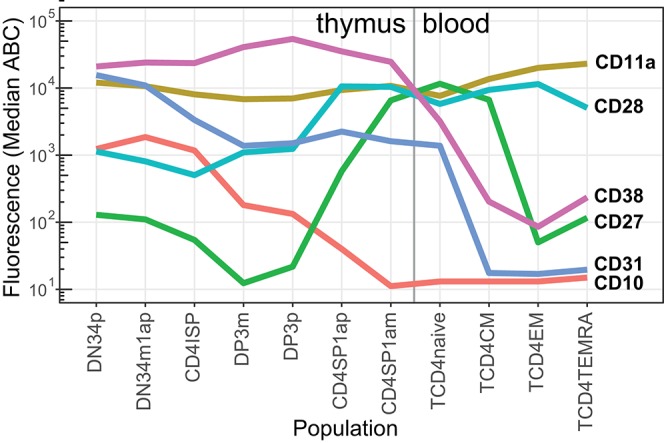
Median expression levels of critical CD markers during thymocyte differentiation into peripheral CD4 T-cells. Expression levels (in ABC) of selected CD markers (*n* = 6) on thymocyte (red labels) and blood (pink labels) CD4 T-cell subsets (*n* = 11).

In addition to mature T-cells in blood, T-cell progenitors in thymus were examined with a separate tube (Supplementary Figure 3) (29, 42), thereby enabling complete mapping of CD marker expression from early T-cell progenitors until effector memory cells (Figure 7) with the maturation tool in the web resource (Figure 1). This revealed that CD10 is gradually lost as cells differentiate from the double negative (DN) to the double positive (DP) stage, and is completely absent on single positive (SP) CD4+ T-cells. Distinct expression patterns were seen for costimulatory molecules CD27 and CD28. Early progenitors already expressed medium levels of CD28, which increased to a maximum after the DP stage, whereas CD27 was low or absent until the DP stage, reaching its maximum just before thymocytes exit to periphery at the CD1a-SP CD4 stage. All thymocytes expressed CD31, which was gradually lost on peripheral naive CD4 T-cells. CD11a was expressed on all stages of T-cell differentiation, with varying degrees of intensity, and a peak on effector memory T-cells.

### Antigen-Dependent B-Cell Maturation in Tonsil

Within the total HCA of CD1–CD100 on all cell subsets (Figure 4 and Supplementary Figure 8), the tonsil B-cell subsets were clustered together, and within this cluster, three subclusters were formed containing the three major functional compartments: (i) B-lymphocytes, including naive and unswitched and switched memory B-cells; (ii) germinal center (GC) cells, including centrocytes (CC) and centroblasts (CB); and (iii) plasma cells (PC), including CD138– and CD138+ PC. Over 30 CD markers showed statistically significant differences (*p* < 0.01) between any two of these three major subsets, and a *p* < 0.001 was observed for >20 CD markers. Populations within each of the three these subgroups were very homogeneous based with <5 CD markers significantly different (*p* < 0.01) between them.

**Figure 8:**
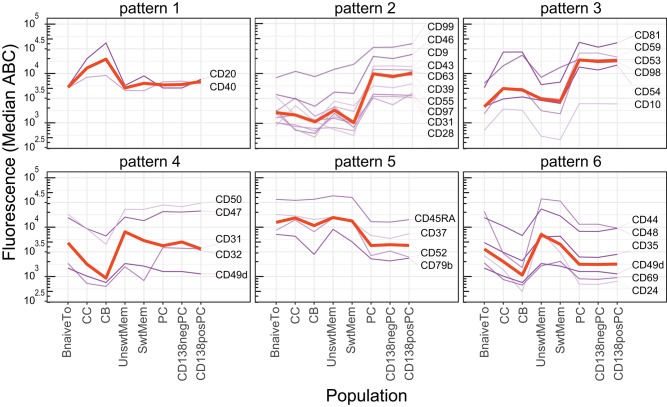
Expression pattern of selected CD markers during B-cell maturation in tonsil. Expression patterns (median ABC) of CD markers showing statistically significant increased **(Upper)** or decreased **(Lower)** level of expression on germinal center (GC) cells (patterns 1 and 4), plasma cells (PC) (patterns 2 and 5), and both GC + PC simultaneously (patterns 3 and 6), as compared to B-lymphocytes (naive and memory B-cell subsets). Red lines indicate median expression levels of the depicted markers in that panel.

PC and B-lymphocyte groups were most different with in CD marker expression (*p* < 0.01, 37 CD markers; *p* < 0.001, 27 CD markers). Those differences with a *p* < 0.001 included upregulation of a large set of adhesion and signaling molecules (CD18, CD31, CD54, CD97, CD98, and CD99) together with a different profile of expression of activation/signaling markers (CD9, CD24, CD27, CD28, CD37, CD39, CD43, CD44, CD45RA, CD52, CD53, CD63, CD79b, and CD81) and complement receptor proteins (CD35, CD46, CD55, and CD59) (28, 43). Visualization with the maturation tool from the CD Maps web resource (Figure 1) showed that some of these phenotypic features of an antibody-secreting cell signature were already acquired in the GC compartment (Figure 8). These phenotypic changes included upregulation of molecules involved in adhesion/migration (CD54, CD98) and enzymatic activity (CD10; pattern 3); changes in cell activation/signaling (CD24, CD44) and complement receptors (CD35, CD59; pattern 6), as compared to B-lymphocytes. PC and GC groups differed in 20 CD markers (*p* < 0.001), including those that were already upregulated during the GC phase (CD54, CD59, and CD98; pattern 3), reversion of phenotypic changes observed during GC reaction (CD20, CD31, CD32, CD40, CD47, and CD55; patterns 1, 2, and 4), and upregulation of markers that were absent on both B-lymphocytes and GC cells (CD9, CD28, CD43, CD63, and CD97). Finally, some markers were upregulated (CD46 and CD99; pattern 2) or decreased as compared to both B-lymphocytes and GC (CD37, CD45RA, and CD52; pattern 5).

## Discussion

We here examined 111 CD markers on 47 leukocyte subsets using multicolor flow cytometry with the marker of interest in the PE channel. The resulting expression profile is the largest quantitative dataset of surface protein expression levels on human immune cells.

The examined surface proteins represent those that were defined clustered mAbs in HLDA workshops I–V that were held in the 1980s and early 1990s (20–24). At that time, the protein expression patterns were defined in great detail. However, with advances in technologies and new insights into immune cell function and subsets, we deemed the expression data incomplete, not fully accurate, and lacking quantitative information. Indeed, when we compare our data with a CD chart of a major antibody vendor, we could find over 50 discrepancies and 25 missing values. In part, those discrepancies stem from a positivity and negativity definition on a broadly defined cell lineage: any positivity found at any stage and/or activation status is regarded as positivity on such chart. Our detailed analysis on well-defined subsets potentially clarifies this.

To ensure robustness and reproducibility of our data, we standardized our experimental procedures and flow cytometer setup according to the protocols that were established for clinical use by the EuroFlow consortium (www.EuroFlow.org) (14). Subsequently, the measurements were independently performed in three to four laboratories, each acquiring data from three to four donors with parallel acquisition of PE signal calibration particles. Indeed, gating of subsets using the backbone markers could be reliably performed on the data, irrespective of their origin. There are limitations in the interpretation of the signal near the background (a combination of autofluorescence, spillover spread, non-specific antibody binding, and antibody titer) that resulted in a “gray zone” at 200–700 ABC units in lymphocytes and 1,000–10,000 ABC units in myeloid cells that has to be evaluated by a more sensitive approach in future studies.

Thus, we have obtained a realistic dataset, which can be prepared reproducibly in any laboratory following the same operating procedure. Although we do not claim we have covered population variation with only 12 donors per CD marker, by displaying up to 12 donors using median values, outliers caused by, e.g., rare genetic polymorphisms (CD45 isoforms or CD39) or by accidental activation (CD69) would not overtly affect the results (44–46). Accurate quantification of CD marker expression levels is not only important for biological function, but can be utilized as well for a proper design of flow cytometry experiments, where also intensity of expression is essential information for a successful multicolor panel (47).

The unique feature of our data resource is the detailed information in expression levels and changes between diverse immune cell subsets, thus allowing interpretation of quantitative changes during thymocyte development, B-cell maturation in the tonsil, and between blood cell subsets that might share expression of the same marker but with different quantities.

In the present study, we quantitatively mapped the expression of 111 surface-expressed proteins on 41 non-overlapping leukocyte subsets from three human tissues. With this being a large-scale analysis and a systems approach, a few concessions had to be made in experimental design. Accuracy of exact quantification of CD marker expression is potentially skewed by the antibody binding occurring through either one or two Fab domains (48). Thus, the ABC unit that was used to quantitatively depict expression has an error margin of a factor 2 for the number of expressed molecules. Still, our measurements for CD4 yielded a median of 38,650 ABC (clone MEM-241) for naive CD4 T-cells, which was very similar to the previously published value of 42,000 ABC (clone SK3) (49). Finally, for this large-scale approach, we only could use one antibody reagent for each given CD marker. Selection criteria for these reagents included (1) being a clone that was approved in the HLDA workshops and (2) good reactivity based on our in-house experience. Our pilot tests for two clones for CD4 (MEM-241 and RPA-T4) and CD8 (MEM-31 and HIT8a) showed differences of up to 20% in expression levels. As the clones we tested have been through the HLDA workshops, these will serve as a benchmark that can either be matched or can be surpassed by alternative reagents. The resource we have built will be appended in the future with new clones, new reagents, new CD markers, and new cell subsets. In the upcoming 11th HLDA workshop, this methodological framework will be used to measure and cluster antibody reactivities across subsets to help assign new CD nomenclature. This approach follows the strategy proposed by the International Working Group for Antibody Validation (IWGAV) that has documented expression patterns for 3,706 antibodies in immunoprecipitates (50, 51). Including future reactivity patterns of HLDA 11 in the CD Maps resource will enhance its role as a benchmark for the research community.

Regarding the immunobiology, we did not exhaustively define all functionally defined immune cell subsets. With four tubes using seven channels for the backbone each, we were able to define 41 unique, non-overlapping subsets. Several cell types were not included, such as helper T-cell subsets, regulatory T-cells, NK T-cells, and mucosa-associated invariant T-cells (MAIT). With an extended panel using more fluorescent markers, such limitation can be overcome in future studies. However, rare cell populations such as innate lymphoid cells will remain a challenge as this would require the acquisition of more than a million events per staining.

In conclusion, we have demonstrated the possibility to systematically quantify the expression of surface-expressed proteins on the multitude of immune cells using standardized multicolor flow cytometry. There is a need for this standardized systems approach to avoid confusion from separate observations in individual laboratories, to correct potential mistakes in the literature, and to predict potential off-target effects of antibody-based therapies. The CD Maps web resource enables each user to explore the data and it has the capacity to function as a platform for surface molecule expression data that can be updated with newer CD markers and more leukocyte subsets. With the ongoing activities of the HLDA workshops, the CD Maps project can provide the means to get toward a full picture of the surfaceome of human immune cells.

## Data Availability Statement

The datasets generated for this study can be found in the CD Maps web resource: http://www.hcdm.org.

## Ethics Statement

The studies involving human participants were reviewed and approved by the use of human pediatric tissue and adult buffy coats was approved by the Human Ethics Committees of the Erasmus Medical Center, the University Hospital Motol, and the universities of Salamanca and Barcelona. Written informed consent to participate in this study was provided by the participants' legal guardian/next of kin.

## Author Contributions

TK, MP-A, PE, and MZ conceptualized the study and designed the experiments. DK, MC, SB, and EB performed the experimental work. KF integrated all data, performed bioinformatics analysis, and built the online web resource. TK, KF, MP-A, PE, and MZ wrote the paper. All authors performed data analysis, commented on draft versions and approved the final manuscript.

### Conflict of Interest

The authors declare that the research was conducted in the absence of any commercial or financial relationships that could be construed as a potential conflict of interest.
