# Supplementary material for: CD Maps—Dynamic Profiling of CD1–CD100 Surface Expression on Human Leukocyte and Lymphocyte Subsets
Source: Front Immunol. 2019 Oct 23;10:2434. doi: 10.3389/fimmu.2019.02434 (PMC6820661; doi:10.3389/fimmu.2019.02434)
Supplement: Supplementary file 1 [file Image_1.pdf]

Suppl Figure 1.

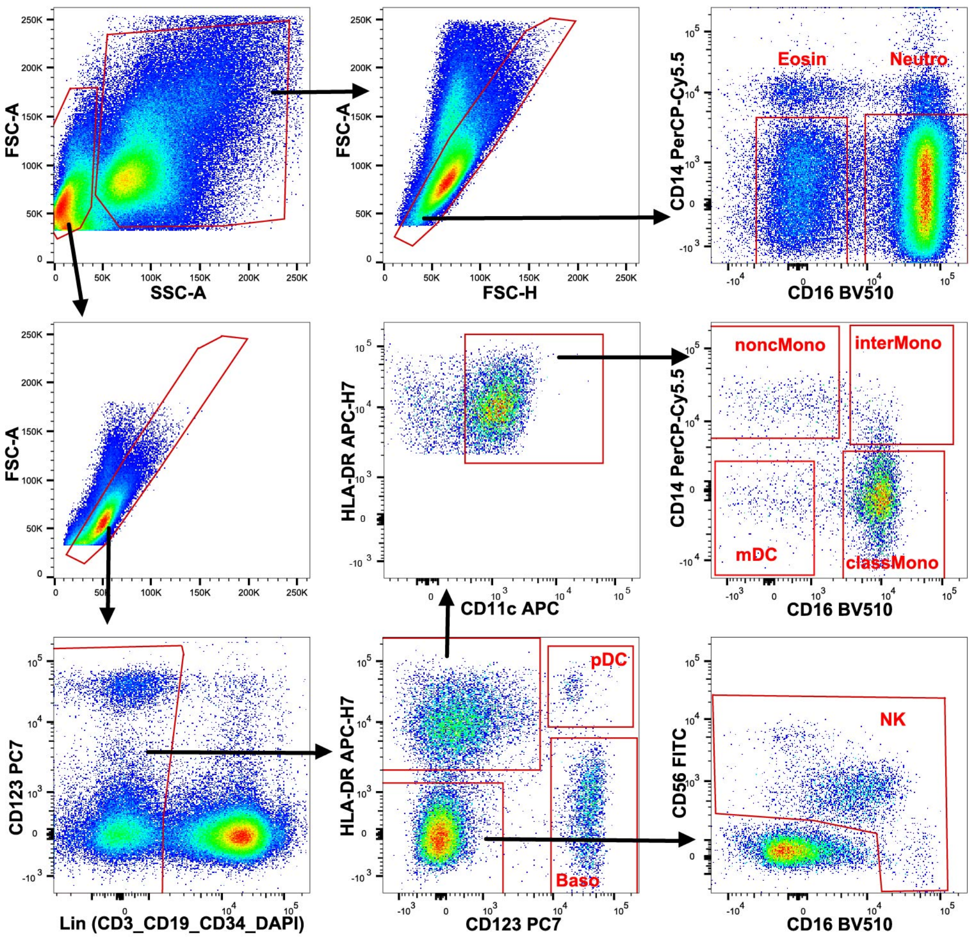

Gating strategy and definition of innate cell types in blood. Eosinophils and neutrophils were defined within the $FSC^{hi}SSC^{hi}$ gate and following exclusion of doublets, gated on the $CD14^{-}$ $CD16^{dim}$ (eosin) and $CD14^{-}CD16^{hi}$ (neutro) phenotypes. Within the $FSC^{dim}SSC^{dim}$ gate, single cells were selected and expression of lineage markers and DAPI was excluded, prior to definition of $CD123^{+}$HLA-DR^{-} basophils and $CD123^{+}$HLA-DR^{+} plasmacytoid (p)DCs. NK cells were defined as CD16 and/or $CD56^{+}$ within the $CD123^{-}$HLA-DR^{-} gate. Finally, HLA-$DR^{+}CD11c^{+}$ cells were subdivided into $CD14^{+}CD16^{-}$ classical monocytes, $CD14^{+}CD16^{+}$ intermediate monocytes, $CD14^{-}CD16^{+}$ non-classical monocytes, and $CD14^{-}CD16^{-}$ DCs.
