# Supplementary material for: CD Maps—Dynamic Profiling of CD1–CD100 Surface Expression on Human Leukocyte and Lymphocyte Subsets
Source: Front Immunol. 2019 Oct 23;10:2434. doi: 10.3389/fimmu.2019.02434 (PMC6820661; doi:10.3389/fimmu.2019.02434)
Supplement: Supplementary file 2 [file Image_2.pdf]

Suppl Figure 2.

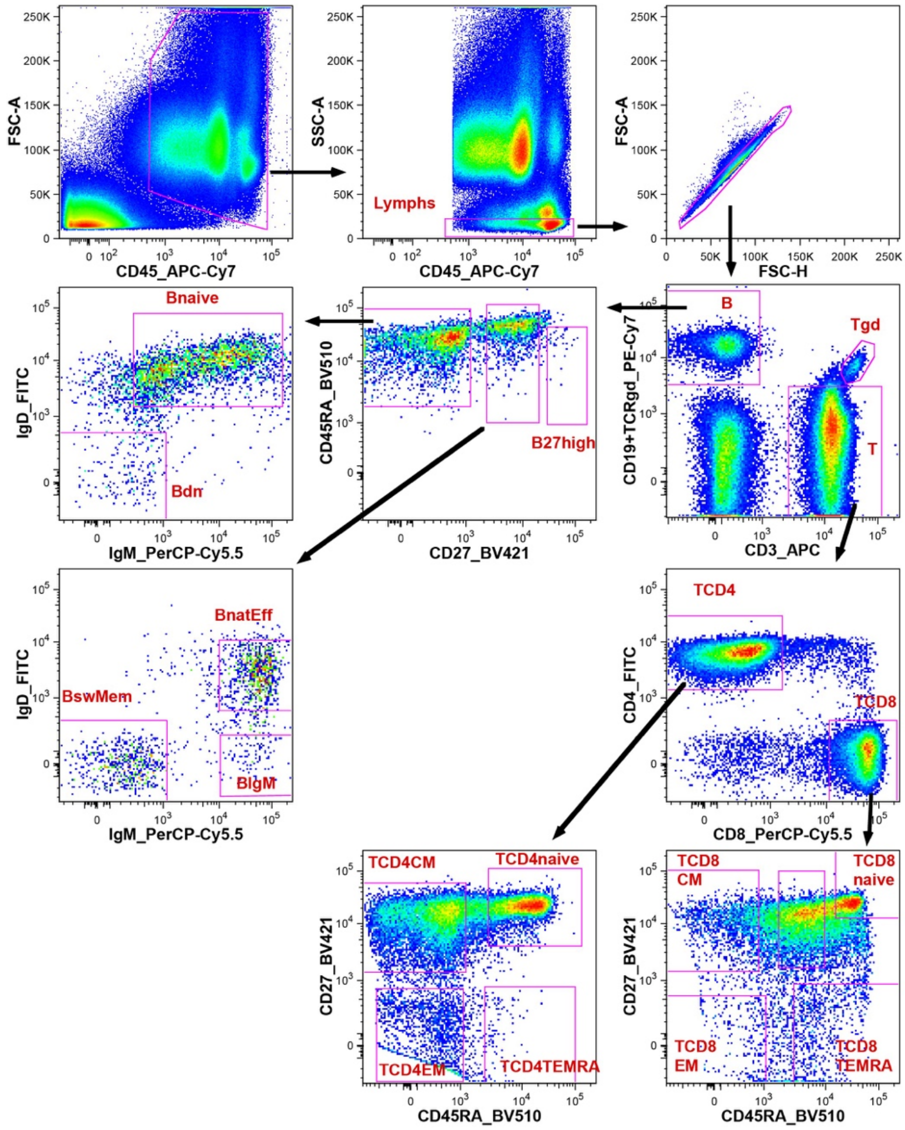

Gating strategy and definition of B- and T-cell subsets in blood. FSC^{dim}SSC^{dim}CD45^{+} lymphocytes were gated prior to exclusion of doublets. Subsequently, CD19^{+} B cells were defined, as well as CD3^{+}TCRγδ^{+} T cells and CD3^{+}TCRγδ^{-} T cells. Within CD19^{+} B cells, the following subsets were defined: CD27^{-}IgM^{+}IgD^{+} naive B cells, CD27^{-}IgM^{-}IgD^{-} B cells, CD27^{+}IgM^{+}IgD^{+} natural effector, CD27^{+}IgM^{+}IgD^{-} IgM-only, and CD27^{+}IgM^{-}IgD^{-} Ig-switched memory B cells. CD3^{+}TCRγδ^{-} T cells were subdivided into the CD4 and CD8 lineages, and within each, CD45RA^{+}CD27^{+} naive, CD45RA^{-}CD27^{+} central memory, CD45RA^{-}CD27^{-} effector memory, and CD45RA^{+}CD27^{-} effector memory RA T cells were defined.
