# Supplementary material for: CD Maps—Dynamic Profiling of CD1–CD100 Surface Expression on Human Leukocyte and Lymphocyte Subsets
Source: Front Immunol. 2019 Oct 23;10:2434. doi: 10.3389/fimmu.2019.02434 (PMC6820661; doi:10.3389/fimmu.2019.02434)
Supplement: Supplementary file 3 [file Image_3.pdf]

Suppl Figure 3.

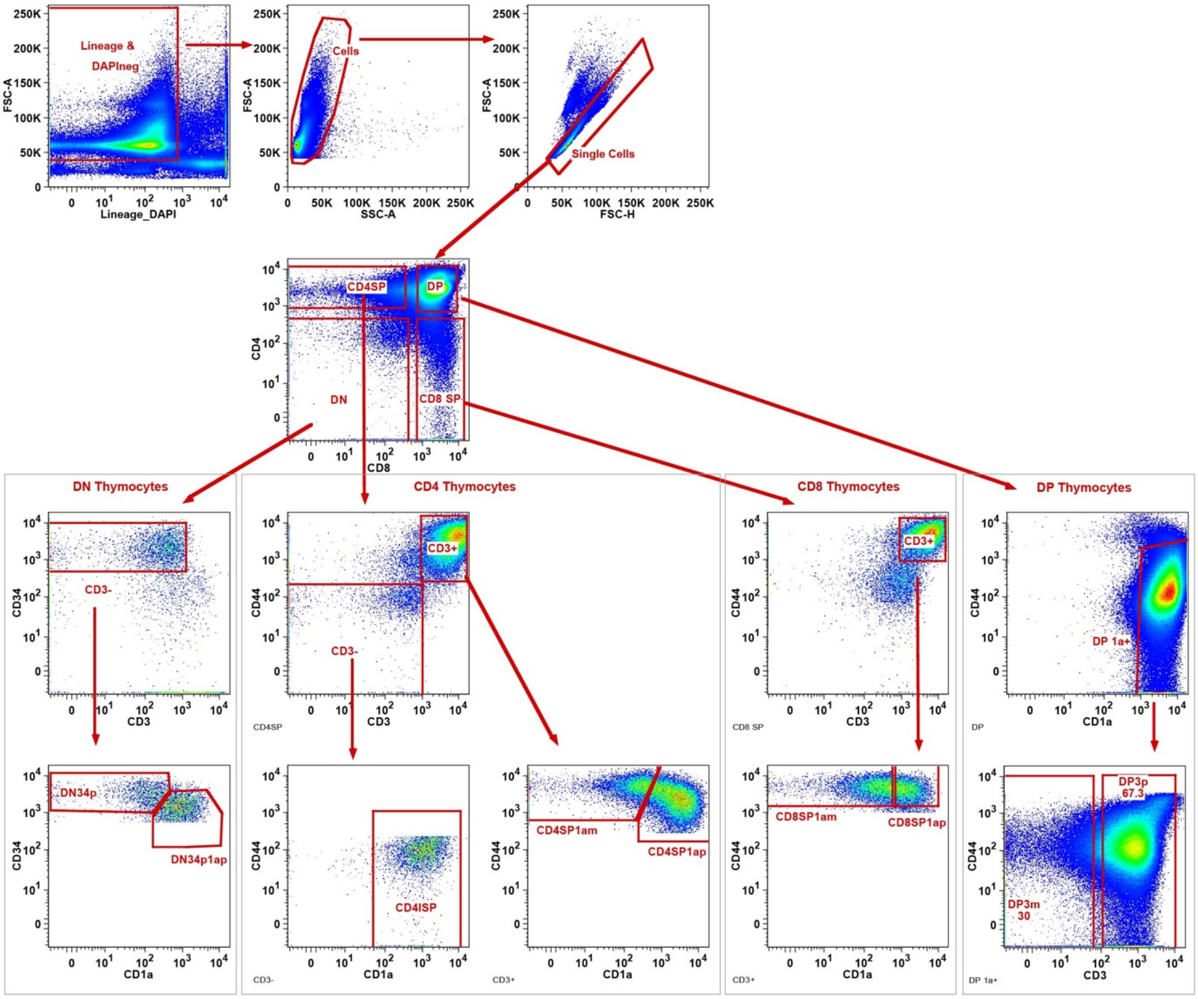

Gating strategy and definition of thymocytes. FSC^{dim}SSC^{dim}DAPI^{-} single thymocytes were selected, prior to subdivision based on CD4 and CD8 into four major stages: double negative (DN), single positive (SP) and double positive (DP). CD3^{-} DN thymocytes were split into the earliest CD34^{+}CD1a^{-} and subsequent CD34+CD1a^{+} subsets.

Within CD4 SP, CD3^{-}CD1a^{+} immature single positive (ISP) thymocytes were defined. Double positive thymocytes were split into the earlier CD3^{-} and later CD3^{+} subsets. Finally, CD3+ SP within both the CD4 and CD8 lineage were separated into the earlier CD1a+ and later CD1a^{+} subsets.
