# Supplementary material for: CD Maps—Dynamic Profiling of CD1–CD100 Surface Expression on Human Leukocyte and Lymphocyte Subsets
Source: Front Immunol. 2019 Oct 23;10:2434. doi: 10.3389/fimmu.2019.02434 (PMC6820661; doi:10.3389/fimmu.2019.02434)
Supplement: Supplementary file 4 [file Image_4.pdf]

Suppl Figure 4.

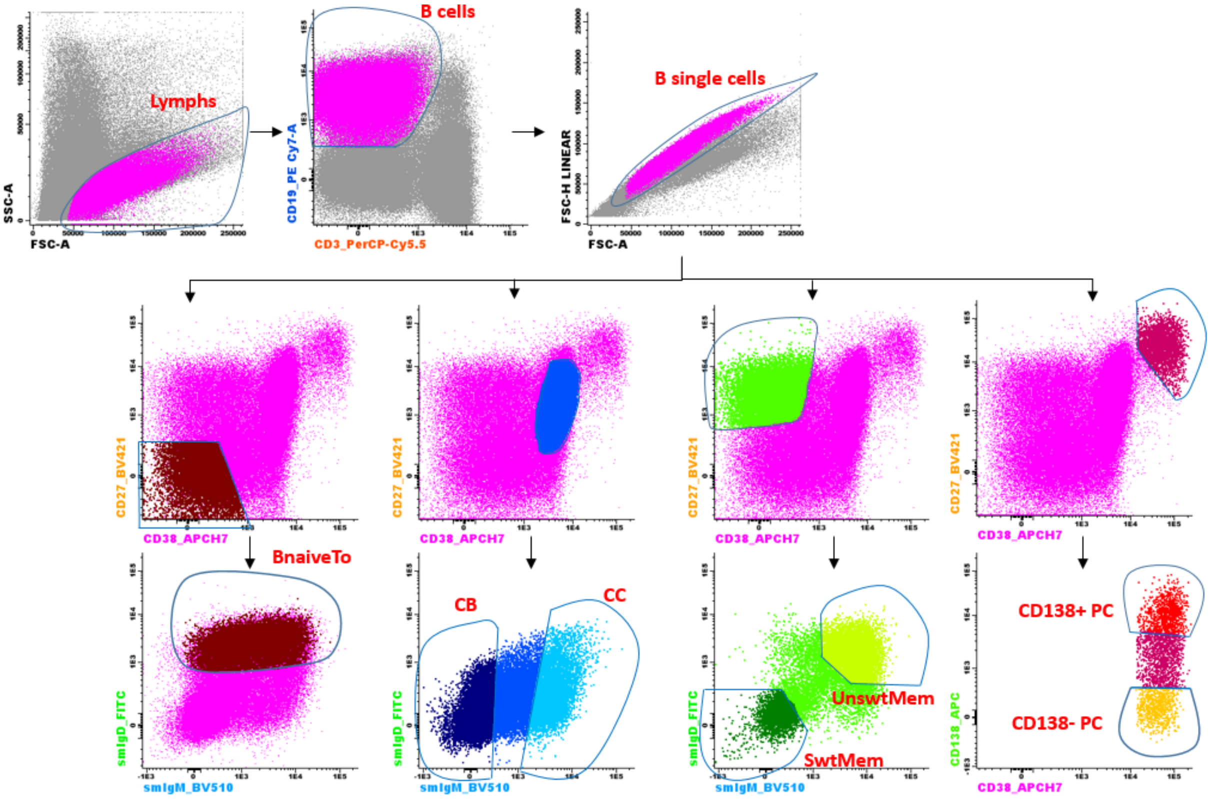

Gating strategy and definition of B cells in tonsil. FSC^{dim}SSC^{dim}CD19^{+} B cells were gated prior to exclusion of doublets. Subsequently, Within CD19^{+} B single cells, the following subsets were defined: CD27^{-}CD38^{-}IgM^{+}IgD^{+} naive B cells Tonsils, CD27^{+}CD38^{+}IgM^{-}IgD^{-} centroblasts, CD27^{+}CD38^{+}IgM^{+}IgD^{-} centrocytes, CD27^{+}CD38^{-}IgM^{-}IgD^{-} switched memory B cells, CD27^{+}CD38^{-}IgM^{+}IgD^{+} unswitched memory B cells, CD27^{++}CD38^{++} CD138^{+} plasma cells and CD27^{++}CD38^{++} CD138^{-} plasma cells.
