# Supplementary figures and images for: CD Maps—Dynamic Profiling of CD1–CD100 Surface Expression on Human Leukocyte and Lymphocyte Subsets
Source: Front Immunol. 2019 Oct 23;10:2434. doi: 10.3389/fimmu.2019.02434 (PMC6820661; doi:10.3389/fimmu.2019.02434)

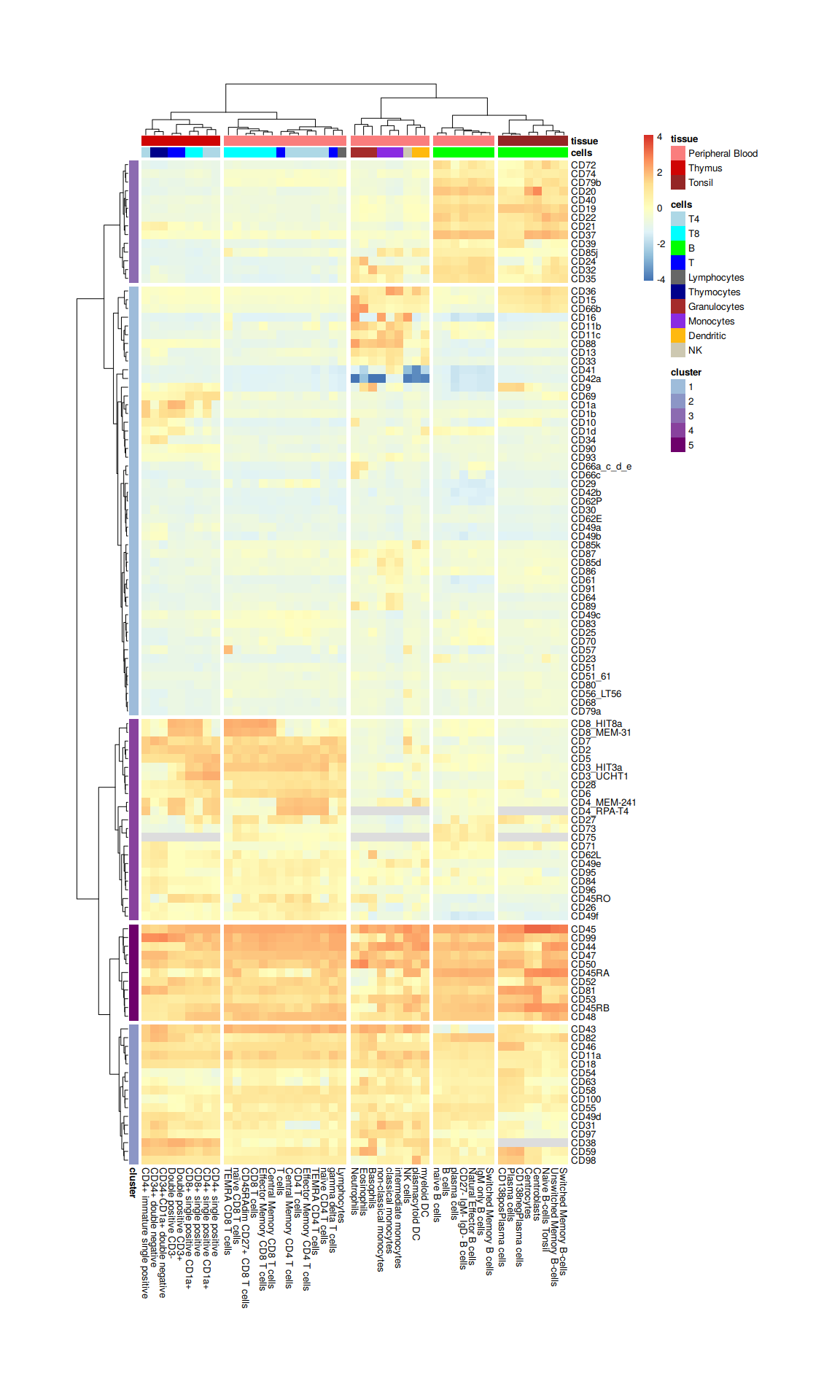

Supplement: Supplementary file 8 [file Image_8.PNG]
