# Supplementary material for: CD Maps—Dynamic Profiling of CD1–CD100 Surface Expression on Human Leukocyte and Lymphocyte Subsets
Source: Front Immunol. 2019 Oct 23;10:2434. doi: 10.3389/fimmu.2019.02434 (PMC6820661; doi:10.3389/fimmu.2019.02434)
Supplement: Supplementary file 9 [file Image_9.pdf]

Supplementary Figure 9.

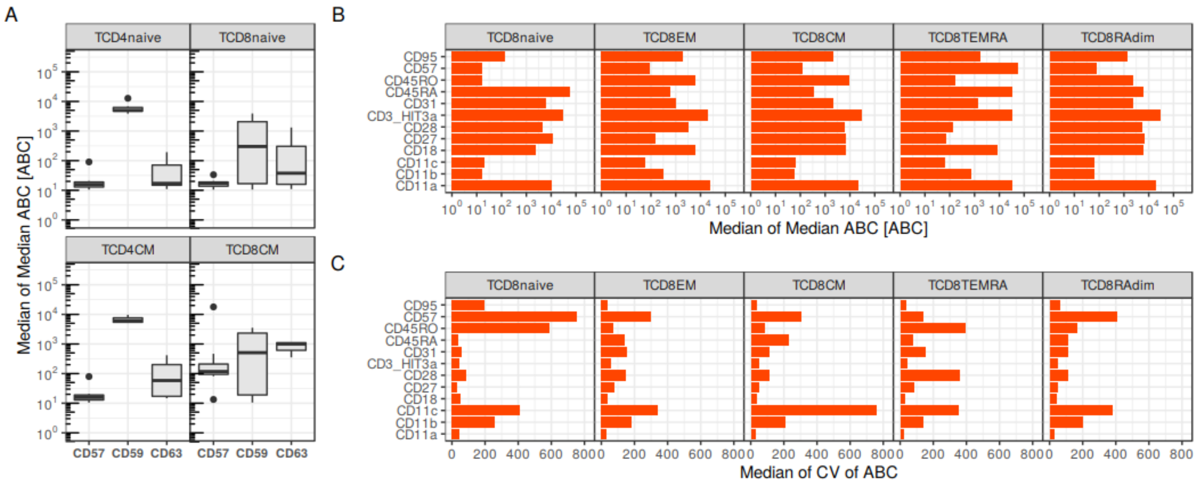

(A) CD4 subsets express higher levels of CD59 than CD8 subsets, while CD8 CM express higher CD57 and CD63 **(B)** Differential expression patterns of multiple markers during CD8 T-cell maturation. Median expression levels of all donors shown. **(C)** Inter-donor variation of expression for markers shown in panel B.
