# Supplementary material for: CD Maps—Dynamic Profiling of CD1–CD100 Surface Expression on Human Leukocyte and Lymphocyte Subsets
Source: Front Immunol. 2019 Oct 23;10:2434. doi: 10.3389/fimmu.2019.02434 (PMC6820661; doi:10.3389/fimmu.2019.02434)
Supplement: Supplementary file 10 [file Table_1.pdf]

Suppl Table 1. Backbone markers flow cytometry panels

tube	marker	clone	fluorochrome	supplier
A. Blood innate				
	CD3	SK7	BV421	BD Biosciences
	CD19	HIB19	BV421	BD Biosciences
	CD34	581	BV421	BD Biosciences
	LIVE/DEAD™ Fixable Violet Dead Cell Stain Kit			Molecular Probes
	CD16	3G8	BV510	BioLegend
	CD56	B159	FITC	BD Biosciences
	CD14	M5E2	PerCP-Cy5.5	BD Biosciences
	CD123	6H6	PE-Cy7	BioLegend
	CD11c	B-ly6	APC	BD Biosciences
	HLA-DR	L243	APC-H7	BD Biosciences
B. Blood adaptive				
	CD27	O323	BV421	BioLegend
	CD45RA	HI100	BV510	BD Biosciences
	CD4	MEM-241	FITC	Exbio
	IgD	IA6-2	FITC	BioLegend
	CD8	MEM-31	PerCP-Cy5.5	Exbio
	IgM	MHM-88	PerCP-Cy5.5	BioLegend
	CD19	LT-19	PC7	Exbio
	TCRγδ	11F4	PE-Cy7	BD Biosciences
	CD3	UCHT-1	APC	Exbio
	CD45	MEM-28	APC-Cy7	Exbio
C. Tonsil B-cells				
	CD27	O323	BV421	BioLegend
	IgM	MHM-88	BV510	BioLegend
	IgD	IA6-2	FITC	BioLegend
	CD3	SK7	PerCP-Cy5.5	BD Biosciences
	CD19	SJ25C1	PE-Cy7	BD Biosciences
	CD138	MI15	APC	BD Biosciences
	CD38	HB7	APC-H7	BD Biosciences
D. Thymocytes				
	CD13	WM15	BV421	BD Biosciences
	CD19	HIB19	BV421	BD Biosciences
	CD33	WM53	BV421	BD Biosciences
	CD16	3G8	BV421	BD Biosciences
	CD56	NCAM16.2	BV421	BD Biosciences
	LIVE/DEAD™ Fixable Violet Dead Cell Stain Kit			Molecular Probes
	CD4	OKT4	BV510	BioLegend
	CD44	G44-26	FITC	BD Biosciences
	CD3	SK7	PerCP-Cy5.5	BD Biosciences
	CD34	8G12	PE-Cy7	BD Biosciences
	CD1a	HI149	APC	BD Biosciences
	CD8	SK1	APC-H7	BD Biosciences
