# Supplementary material for: CD Maps—Dynamic Profiling of CD1–CD100 Surface Expression on Human Leukocyte and Lymphocyte Subsets
Source: Front Immunol. 2019 Oct 23;10:2434. doi: 10.3389/fimmu.2019.02434 (PMC6820661; doi:10.3389/fimmu.2019.02434)
Supplement: Supplementary file 11 [file Table_2.pdf]

Suppl Table 2. CD1-CD100 marker details

CD marker	Clone(s)	Company/-ies	OTHER NAMES	type of molecule	GENE NAME	Molecular weight (kD, Unreduced)	Known or Proposed Function
CD1a	HI149	Exbio	R4, HTA1	IgSF	CD1a	49	Non-peptide antigen presentation
CD1b	SN13	BioLegend	R1	IgSF	CD1b	45	Non-peptide antigen presentation
CD1d	CD1d42	BD	R3	IgSF	CD1d	49	Non-peptide antigen presentation
CD2	RPA-2.10	BD	LFA-2	IgSF	CD2	50	T cell and NK cell activation
CD3	UCHT1, HIT3a	Exbio, BD	T3, Leu4, OKT3	IgSF	CD3E	20	T cell activation
CD4	RPA-T4, MEM-241	BD, Exbio	T4, Leu3a, OKT4	IgSF	CD4	55	T cell activation
CD5	UCHT2	BD	Leu-1	SR	CD5	58	Cell signaling
CD6	M-T605	BD	T12	SR	CD6	100-120 [reduced]	Cell signaling
CD7	M-T701	BD	gp40	IgSF	CD7	38	T cell activation
CD8a	HIT8a, MEM-31	BD, Exbio	T8, Leu2, OKT8	IgSF	CD8A	68; 32-34 [reduced]	T cell activation
CD8b	2ST8-5H7	BD	CD8b	IgSF	CD8B	68; 30-32 [reduced]	T cell activation
CD9	M-L13	BD	p24, MRP-1	TSPAN	CD9	26	T cell activation, platelet activation
CD10	HI10a	BD	CALLA, gp100, NEP	Metalloproteinase	MME	100	Enzymatic activity, B cell differentiation
CD11a	HI111	BD	LFA-1	Integrin	ITGAL	170	Adhesion, migration
CD11b	ICRF-44	BD	Mac-1	Integrin	ITGAM	165	Adhesion, migration
CD11c	B-ly6	BD	p150, ,95α	Integrin	ITGAX	145	Adhesion, migration
CD13	WM15	BD	APN, gp150	Metalloproteinase	ANPEP	150	Enzymatic activity
CD14	M5E2	BD	LPS R	Leucine-rich repeat	CD14	53	Myeloid cell activation
CD15	HI98	BD	Lewis X, Lex	Carbohydrate	–	–	Adhesion, migration
CD16	LNK16	Exbio	CD16a, FcγRIIIA	IgSF	FCGR3A	50-65	Phagocytosis and ADCC
CD18	6.7	BD	β2 integrin	Integrin	ITGB2	120	Adhesion, migration
CD19	HIB19	BD	B4	IgSF	CD19	90	B cell activation
CD20	2H7	BD	B1, Bp35	TM4	MS4A1	33/35/37	B-cell activation
CD21	B-ly4	BD	CR2, EBV-R, C3dR	RCA	CR2	130-145	Complement receptor, B cell activation
CD22	HIB22	BD	BL-CAM, SIgSFlec-2	IgSF	CD22	140	B cell inhibitory receptor
CD23	M-L233	BD	FcεRII, BLAST-2	C-type lectin	FCER2	45	Low-affinity IgE receptor, B cell activation
CD24	ML5	BD	BA-1, HAS	Sialoprotein	CD24	35-45	B cell activation, granulocyte activation
CD25	M-A251	BD	Tac, p55, IL-2Ra	CCP-like	IL2RA	55	Cytokine receptor, lymphocyte activation
CD26	M-A261	BD	Dipeptidyl peptidase IV (DPP IV)	Peptidase	DPPA	110	Enzymatic activity
CD27	M-T271	BD	T14, S152, TNFRSF7	TNFR	TNFRSF7	110-120	Cell signaling

CD28	CD28.2	BD	Tp44, T44	IgSF	CD28	90	T cell activation
CD29	HUTS-21	BD	Integrin b1	Integrin	ITGB1	110	Adhesion, migration
CD30	Ber-H8	BD	Ki-1, TNFRSF8	TNFR	TNFRSF8	120	Cell death
CD31	WM59	BD	PECAM-1, endocam	IgSF	PECAM1	130-140	Adhesion, migration
CD32	3D3	BD	FcγRII	IgSF	FCGR2A	40	Phagocytosis, cell signaling
CD33	WM53	Exbio	p67, Siglec-3	IgSF	CD33	150	Myeloid inhibitory receptor
CD34	4H11[APG]	Exbio	gp10-120, Mucosialin, MY10	Sialomucin	CD34	105-120	Adhesion
CD35	E11	BD	CR1, C3b/C4b-R	RCA	CR1	165-255	Complement receptor
CD36	CB38	BD	GPIV, gpIIIb	SR	CD36	88-113	Pathogen recognition
CD37	M-B371	BD	gp 52-40	TSPAN	CD37	40-52	Cell signaling
CD38	HIT2	Exbio	T10, ADP-ribosyl cyclase	ectoenzyme	CD38	45	Enzymatic activity, cell signaling
CD39	TU66	BD	Entpd1, NTPDase-1	ectoenzyme	ENTPD1	78	Enzymatic activity, cell signaling
CD40	5C3	BD	TNFRSF5	TNFR	TNFRSF5	85 (dimers)	B cell activation, monocyte and DC activation
CD41	MEM-06	Exbio	GPIIb	Integrin	ITGA2B	120/23	Adhesion, platelet aggregation
CD42a	ALMA.16	BD	GPIX	LRR	GP9	22	Platelet adhesion
CD42b	HIP1	BD	GPIBα	LRR	GP1BA	160	Platelet adhesion
CD43	1G10	BD	sialophorin,leukosialin	Sialomucin	SPN	95-135	Cell signaling
CD44	G44-26	BD	HCAM, Pgp-1	Hyaladhesin	CD44	85	Cell signaling
CD45	HI30	BD	LCA, T200	RPTP	PTPRC	180-240	Cell signaling
CD45RA	MEM-56	Exbio	Leu18	RPTP	CD45 isoform	205-220	Cell signaling
CD45RB	MT4	BD		RPTP	CD45 isoform	190-220	Cell signaling
CD45RO	UCHL1	BD	UCHL-1	RPTP	CD45 isoform	180	Cell signaling
CD46	MEM-258	Exbio	MCP	RCA	MCP	52-58	Complement regulation
CD47	B6H12	BD	Integrin-Associated Protein	IgSF	CD47	45-60	Adhesion, cell signaling
CD48	Tü145	BD	BLAST1, BCM1, SLAMF2	IgSF	CD48	45	Cell activation
CD49a	SR84	BD	VLA4-1a, α1 integrin	Integrin	ITGA1	200	Adhesion, migration
CD49b	12F1	BD	VLA4-2a, α2 integrin	Integrin	ITGA2	160	Adhesion, migration
CD49c	C3 II.1	BD	VLA4-3a, α3 integrin	Integrin	ITGA3	150	Adhesion, migration

CD49d	9F10	BD	VLA4-4a, α4 integrin	Integrin	ITGA4	150/180	Adhesion, migration, lymphocyte development
CD49e	NKI-SAM-1	BioLegend	VLA4-5a, α5 integrin	Integrin	ITGA5	155	Adhesion, migration
CD49f	GOH3	BD	VLA4-6a, α6 integrin	Integrin	ITGA6	140	Adhesion, migration
CD50	CBR IC3/1	BioLegend	ICAM-3	IgSF	ICAM3	110-170	Adhesion, cell signaling
CD51	NKI-M9	BioLegend	Vitronectin R	Integrin	ITGAV	150	Adhesion, migration
CD51/61	23C6	BioLegend	Vitronectin R / gpIIIa, b3 integrin	Integrin	ITGAV / ITGB3	150 / 90	Adhesion, migration / platelet aggregation
CD52	HI186	BioLegend	CAMPATH-1	Sialoprotein	CD52	25-29	Cell signaling
CD53	HI29	BioLegend	TSPAN2	TSPAN	CD53	32-42	Cell signaling
CD54	HA58	BioLegend	ICAM-1	IgSF	ICAM1	80-114	Adhesion, migration
CD55	JS11	BioLegend	DAF	RCA	DAF	50/70	Complement regulation
CD56	LT56	BioLegend	NCAM	IgSF	NCAM1	130/140/180	Cell activation
CD57	NK-1	BD	HNK-1, 3-0-sulfated glucuronic acid	Carbohydrate	–	–	Adhesion
CD58	1C3 (AICD58.6)	BD	LFA-3	IgSF	CD58	40-70	T cell and NK cell activation
CD59	p282	BioLegend	Protectin H19	Ly-6	CD59	18-25	Complement regulation
CD61	VI-PL2	BioLegend	gpIIIa, b3 integrin	Integrin	ITGB3	90	Adhesion, platelet aggregation
CD62E	68-5H11	BD	E-selectin, ELAM-1	C-type lectin	SELE	107-115	Adhesion, migration
CD62L	DREG-56	BioLegend	L-selectin	C-type lectin	SELL	65	Adhesion, migration
CD62P	AK-4	BioLegend	P-selectin	C-type lectin	SELP	120	Adhesion, migration
CD63	H5C6	BioLegend	LAMP-3, LIMP, MLA1	TSPAN	CD63	40-60	Cell signaling
CD64	10.1	BioLegend	FcγRI, FcRI	IgSF	FCGR1A	72	Phagocytosis and ADCC
CD66a/c/d/e	B1.1	BD	BGP, CEACAM1, NCA-160	IgSF	CEACAM1	140-180	Adhesion, cell signaling
CD66b	G10F5	BioLegend	NCA-95, CGM6	IgSF	CEACAM8	95-100	Adhesion, cell signaling
CD66c	B6.2	BD	NCA	IgSF	CEACAM6	90 [reduced]	Adhesion, cell signaling
CD68	Y1/82a	BioLegend	macrosialin, gp110	Sialomucin	CD68	110	Pathogen recognition
CD69	FN50	BioLegend	AIM, VEA	C-type lectin	CD69	60	Cell signaling
CD70	Ki-24	BD	Ki-24, CD27L, TNFSF7	TNF	TNFSF7	170 [trimeric]	T cell activation
CD71	Cy IG4	BioLegend	TfR, T9, transferrin receptor	Transferrin	TFRC	190	Metabolism, cell growth
CD72	3F3	Exbio	Lyb-2	C-type lectin	CD72	43	Inhibitory receptor

CD73	AD2	BioLegend	ECTO-5'Nucleotidase	Ectoenzyme	NT5E	69-72	Enzymatic activity
CD74	LN2	BioLegend	li, invariant chain	[no family assigned]	CD74	33-41	Antigen presentation, cell signaling
CD79a	HM47	BioLegend	Igα, MB1	IgSF	CD79A	33	B cell activation
CD79b	CB3-1	BioLegend	Igβ, B29	IgSF	CD79B	31	B cell activation
CD80	2D10	BioLegend	B7, B7-1, BB1	IgSF	CD80	60	Cell activation
CD81	5A6	BioLegend	TAPA-1	TSPAN	CD81	26	Cell activation
CD82	ASL-24	BioLegend	R2,4F9, C33	TSPAN	KAI1	45-90	Cell activation
CD83	HB15e	BioLegend	HB15	IgSF	CD83	43 [reduced]	Cell activation
CD84	CD84.1.21	BioLegend	SLAMF5	IgSF	CD84	68-80	Cell activation
CD85d	42D1	BioLegend	LIR-2, ILT4, LILRB2	IgSF	LILRB2	110	Inhibitory receptor
CD85j	GHI/75	BioLegend	LIR-1, ILT2	IgSF	LILRB1	110	Inhibitory receptor
CD85k	ZM4.1	BioLegend	LIR-5, ILT3	IgSF	LILRB4	60	Inhibitory receptor
CD86	IT2.2	BioLegend	B70, B7-2	IgSF	CD86	80 [reduced]	Cell activation
CD87	VIM5	BioLegend	uPAR, urokinase receptor	Ly-6	PLAUR	35-68	Adhesion, migration
CD88	S5/1	BioLegend	C5aR	TM7	C5R1	43	Complement receptor
CD89	A59	BioLegend	IgA R, FcαR	IgSF	FCAR	45-100	Phagocytosis
CD90	5.00E+10	BioLegend	Thy-1	IgSF	THY1	25-35	Cell signaling
CD91	A2MRa-2	BD	LRP, a2M-R	LDLR	LRP1	600	Phagocytosis, metabolism
CD93	VIMD2	BioLegend	CDw93, C1qR1, GR11	C-type lectin	CD93	110	Complement receptor
CD95	DX2	BioLegend	TNFRSF6, Fas, APO-1	TNFR	TNFRSF6	45	Apotosis
CD96	NK92.39	BioLegend	TACTILE, EMR1, BL-KDD/F12	IgSF	CD96	160/180/240	T cell and NK cell activation
CD97	VIM3b	BioLegend	EMR1	TM7	CD97	28,74-89	Adhesion, migration
CD98	MEM-108	Exbio	FRP-1, 4F2	Solute carrier	SLC3A2	125	Adhesion, metabolism
CD99	HCD99	BioLegend	MIC2, E2	Sialomucin	CD99	32	Adhesion, cell signaling
CD100	A8	BioLegend	SEMA4D	Semaphorin	SEMA4D	300	Cell signaling
