# Supplementary material for: CD Maps—Dynamic Profiling of CD1–CD100 Surface Expression on Human Leukocyte and Lymphocyte Subsets
Source: Front Immunol. 2019 Oct 23;10:2434. doi: 10.3389/fimmu.2019.02434 (PMC6820661; doi:10.3389/fimmu.2019.02434)
Supplement: Supplementary file 12 [file Table_3.pdf]

Suppl Table 3. Naming and phenotypic definitions of leukocyte and lymphocyte subsets

	Short name	Full name	Phenotypic definition *	Flow panel
1	Neutro	Neutrophils	$FSC^{hi}SSC^{hi}CD16^{hi}CD14^{-}$	A
2	Eosin	Eosinophils	$FSC^{hi}SSC^{hi}CD16^{dim}CD14^{-}$	A
3	Baso	Basophils	$FSC^{dim}SSC^{dim}lin2^{-}HLA\text{-}DR^{-}CD123+$	A
4	classMono	classical monocytes	$FSC^{dim}SSC^{dim}lin2^{-}CD11c^{+}HLA\text{-}DR^{+}CD14^{+}CD16^{-}$	A
5	interMono	intermediate monocytes	$FSC^{dim}SSC^{dim}lin2^{-}CD11c^{+}HLA\text{-}DR^{+}CD14^{+}CD16^{+}$	A
6	noncMono	Non-classical Monocytes	$FSC^{dim}SSC^{dim}lin2^{-}CD11c^{+}HLA\text{-}DR^{+}CD14^{-}CD16^{+}$	A
7	NK	NK cells	$FSC^{dim}SSC^{dim}lin2^{-}CD16/CD56^{+}$	A
8	mDC	myeloid DC	$FSC^{dim}SSC^{dim}lin2^{-}CD11c^{+}HLA\text{-}DR^{+}CD14^{-}CD16^{-}$	A
9	pDC	plasmacytoid DC	$FSC^{dim}SSC^{dim}lin2^{-}HLA\text{-}DR^{+}CD123^{+}$ $CD14^{-}CD11c^{-}$	A
10	Lymphs	Lymphocytes	$FSC^{dim}SSC^{dim}$CD45+	B
11	∟T	T cells	$FSC^{dim}SSC^{dim}$CD45+CD3+	B
12	∟Tgd	gamma delta T cells	$FSC^{dim}SSC^{dim}$CD45+CD3+TCRgd+	B
13	∟TCD4	CD4 T cells	$FSC^{dim}SSC^{dim}$CD45+CD3+CD4+	B
14	∟TCD4naive	naive CD4 T cells	$FSC^{dim}SSC^{dim}$CD45+CD3+CD4+CD45RA+CD27+	B
15	∟TCD4CM	Central Memory CD4 T cells	$FSC^{dim}SSC^{dim}$CD45+CD3+CD4+CD45RA-CD27+	B
16	∟TCD4EM	Effector Memory CD4 T cells	$FFSC^{dim}SSC^{dim}$CD45+CD3+CD4+CD45RA-CD27-	B
17	∟TCD4TEMRA	TEMRA CD4 T cells	$FSC^{dim}SSC^{dim}$CD45+CD3+CD4+CD45RA-CD27+	B
18	∟TCD8	CD8 T cells	$FSC^{dim}SSC^{dim}$CD45+CD3+CD8+	B
19	∟TCD8naive	naive CD8 T cells	$FSC^{dim}SSC^{dim}$CD45+CD3+CD8+CD45RA+CD27+	B
20	∟TCD8CM	Central Memory CD8 T cells	$FSC^{dim}SSC^{dim}$CD45+CD3+CD8+CD45RA-CD27+	B
21	∟TCD8EM	Effector Memory CD8 T cells	$FSC^{dim}SSC^{dim}$CD45+CD3+CD8+CD45RA-CD27-	B
22	∟TCD8TEMRA	TEMRA CD8 T cells	$FSC^{dim}SSC^{dim}$CD45+CD3+CD8+CD45RA-CD27+	B
23	∟TCD8RAdim	CD45RAdim CD27+ CD8 T cells	$FSC^{dim}SSC^{dim}$CD45+CD3+CD8+CD45RAdimCD27+	B
24	∟B	B cells	$FSC^{dim}SSC^{dim}$CD45+CD19+	B
25	∟Bnaive	naive B cells	$FSC^{dim}SSC^{dim}$CD45+CD19+CD27-IgM+IgD+	B
26	∟BnatEff	Natural Effector B cells	$FSC^{dim}SSC^{dim}$CD45+CD19+CD27+IgM+IgD+	B
27	∟BIgM	IgM-only memory B cells	$FSC^{dim}SSC^{dim}$CD45+CD19+CD27+IgM+IgD-	B
28	∟BswMem	Ig-switched Memory B cells	$FSC^{dim}SSC^{dim}$CD45+CD19+CD27+IgM-IgD-	B

29	∟Bdn	CD27- IgM- IgD- B cells	$FSC^{dim}SSC^{dim}CD45+CD19+CD27-IgM-IgD-$	B
30	∟B27high	plasma cells	$FSC^{dim}SSC^{dim}CD45+CD19+CD27++$	B
31	∟BnaiveTo	Naive B-cells Tonsil	FSC^{dim} SSC^{dim} $CD19^{+}$ $CD3^{-}$ $CD27^{-}$ $CD38^{-}IgM^{+}IgD^{+}$	C
32	∟CC	Centrocytes	FSC^{dim} SSC^{dim} $CD19^{+}$ $CD3^{-}$ $CD38^{+}IgM^{+}IgD^{-}$	C
33	∟CB	Centroblasts	FSC^{dim} SSC^{dim} $CD19^{+}$ $CD3^{-}$ $CD38^{+}IgM^{-}IgD^{-}$	C
34	∟UnswtMem	Unswitched Memory B-cells	FSC^{dim} SSC^{dim} $CD19^{+}$ $CD3^{-}$ $CD27^{+}$ $CD38^{-}IgM^{+}IgD^{+}$	C
35	∟SwtMem	Switched Memory B-cells	FSC^{dim} SSC^{dim} $CD19^{+}$ $CD3^{-}$ $CD27^{+}$ $CD38^{-}IgM^{-}IgD^{-}$	C
36	∟PC	Plasma cells	FSC^{dim} SSC^{dim} $CD19^{+}$ $CD3^{-}$ $CD27^{hi}$ $CD38^{hi}$	C
37	∟CD138negPC	CD138negPlasma cells	FSC^{dim} SSC^{dim} $CD19^{+}$ $CD3^{-}$ $CD27^{hi}$ $CD38^{hi}$ $CD138^{-}$	C
38	∟CD138posPC	CD138posPlasma cells	FSC^{dim} SSC^{dim} $CD19^{+}$ $CD3^{-}$ $CD27^{hi}$ $CD38^{hi}$ $CD138^{+}$	C
39	DN34p	CD34+ double negative	$FSC^{dim}SSC^{dim}$Lin1–DAPI-CD4-CD8-CD3-CD34+CD1a-	D
40	DN34m1ap	CD34-CD1a+ double negative	$FSC^{dim}SSC^{dim}$Lin1–DAPI-CD4-CD8-CD3-CD34+CD1a+	D
41	CD4ISP	CD4+ immature single positive	$FSC^{dim}SSC^{dim}$Lin1–DAPI-CD4+CD8-CD3-CD44-CD1a+	D
42	DP3m	Double positive CD3-	$FSC^{dim}SSC^{dim}$Lin1–DAPI-CD4+CD8+CD3-	D
43	DP3p	Double positive CD3+	$FSC^{dim}SSC^{dim}$Lin1–DAPI-CD4+CD8+CD3+	D
44	CD4SP1ap	CD4+ single positive CD1a+	$FSC^{dim}SSC^{dim}$Lin1–DAPI-CD4+CD8-CD3+CD44+CD1a+	D
45	CD4SP1am	CD4+ single positive	$FSC^{dim}SSC^{dim}$Lin1–DAPI-CD4+CD8-CD3+CD44+CD1a-	D
46	CD8SP1ap	CD8+ single positive CD1a+	$FSC^{dim}SSC^{dim}$Lin1–DAPI-CD4-CD8+CD3+CD44+CD1a+	D
47	CD8SP1am	CD8+ single positive	$FSC^{dim}SSC^{dim}$Lin1–DAPI-CD4-CD8+CD3+CD44+CD1a-	D

* Lin1 = CD13/CD19/CD33/CD16/CD56; Lin2 = CD19/CD3/CD34
