# Supplementary material for: CD Maps—Dynamic Profiling of CD1–CD100 Surface Expression on Human Leukocyte and Lymphocyte Subsets
Source: Front Immunol. 2019 Oct 23;10:2434. doi: 10.3389/fimmu.2019.02434 (PMC6820661; doi:10.3389/fimmu.2019.02434)
Supplement: Supplementary file 13 [file Table_4.pdf]

Suppl Table 4: R packages used for data import and processing

package	version	reference
d3heatmap	0.6.1.2	Cheng, J. & Galili, T. *D3heatmap: Interactive heat maps using 'htmlwidgets' and 'd3.js'*. (2018).
dendextend	1.9.0	Galili, T. Dendextend: An r package for visualizing, adjusting, and comparing trees of hierarchical clustering. Bioinformatics (2015). doi:10.1093/bioinformatics/btv428
dplyr	0.7.8	Wickham, H., François, R., Henry, L. & Müller, K. *Dplyr: A grammar of data manipulation*. (2018).
ggplot2	3.1.0	Wickham, H. *Ggplot2: Elegant graphics for data analysis*. (Springer-Verlag New York, 2016).
htmlwidgets	1.3	Vaidyanathan, R., Xie, Y., Allaire, J., Cheng, J. & Russell, K. *Htmlwidgets: HTML widgets for r*. (2018).
knitr	1.21	Xie, Y. *Knitr: A general-purpose package for dynamic report generation in r*. (2018).<br>Xie, Y. *Dynamic documents with R and knitr*. (Chapman; Hall/CRC, 2015).<br>Xie, Y. in *Implementing reproducible computational research* (eds. Stodden, V., Leisch, F. & Peng, R. D.) (Chapman; Hall/CRC, 2014).
RColorBrewer	1.1.2	Neuwirth, E. *RColorBrewer: ColorBrewer palettes*. (2014).
reshape2	1.4.3	Wickham, H. Reshaping data with the reshape package. *Journal of Statistical Software* **21,** 1–20 (2007).
shiny	1.2.0.9000	Chang, W., Cheng, J., Allaire, J., Xie, Y. & McPherson, J. *Shiny: Web application framework for r*. (2019).
sicegar	0.2.2	Caglar, M. U. & Wilke, C. O. *Sicegar: Analysis of single-cell viral growth curves*. (2017).
webshot	0.5.1	Chang, W. *Webshot: Take screenshots of web pages*. (2018).
