# Supplementary material for: CD Maps—Dynamic Profiling of CD1–CD100 Surface Expression on Human Leukocyte and Lymphocyte Subsets
Source: Front Immunol. 2019 Oct 23;10:2434. doi: 10.3389/fimmu.2019.02434 (PMC6820661; doi:10.3389/fimmu.2019.02434)
Supplement: Supplementary file 16 [file Data_Sheet_1.PDF]

# HLDA – CD Maps standard operating protocol

# Protocol leukocyte isolation without platelets

***(this protocol allows phenotyping blood cells for tube 1 and 2, for tube 4 and 5, mechanically release lymphocytes from thymus or tonsil and start at point 10)***

## Blood extraction with EDTA

1. Dilute buffy coat 1:5 with sterile PBS (PBS – without Ca and Mg plus 2 mM EDTA).
2. Add 20 mL of 4% dextran solution to 20 mL of the diluted blood in a 50 mL tube. (2 tubes if 40 mL of blood)
3. Mix and let erythrocytes sediment for 30 min.
4. Collect the supernatant carefully (avoid to take erythrocytes) and centrifuge it at 880 rpm for 15 min (no break, 130g).

## Lysis step

1. Remove the supernatant.
2. Add 7,5 mL of 0,2% NaCl solution to the pellet for 55 seconds (keep mixing the cells with the hypotonic solution using a 10 mL pipette)
3. Add 17,5 mL of 1,2% NaCl solution and then PBS to get a final volume of 50 mL.
4. Centrifuge at 880 rpm for 15 min (break = 0, 130g)
5. Repeat the lysis step once more since there are still plenty of erythrocytes.
6. Centrifuge at 880 rpm for 15 min (break = 0, 130g)
7. Resupend cells with PBS + 0.09% NaN3+0.5% BSA+ 1mM EDTA.
8. Count WBC concentration (make a note into protocol Backbone HLDA table)
9. Centrifuge 8min, 500g, RT, remove the supernatant carefully
10. Resupend cells with PBS + 0.09% NaN3+0.5% BSA (final concentration 40 million/mL)

## *Sample staining (work in 96 well plate)*

11. Add Test mAb into each row as per manufacturer recommendation titer (5ul or 10ul)
12. Pipette 40ul of cell (1,6x10^6 cells) suspension into V-bottom 96-well plate
13. Where 5ul (or 2.5ul) of test mAb is recommended, add 5ul (or 7.5ul) of PBS+0.09% of NaN3+0.5% BSA
14. Incubate for 30 min at RT protected from light
15. Prepare reagent mix and add PBS to reach 25ul mix per well (see Backbone table 1 below)
16. Mix reagent mix with pipette and add 25ul of reagent mix into 50ul of cell suspension, mix with pipette.
17. Incubate for 30 min at RT protected from light
18. Add 100 µl PBS+0.09% of NaN3+0.5% BSA
19. Centrifuge 8min, 500g, RT, dump to sink
20. Resuspend the cell pellet in 200 µl PBS+0.09% of NaN3+0.5% BSA.
21. Centrifuge 8min, 500g, RT, dump to sink
22. Resuspend the cell pellet in 200 µl PBS+2mmol EDTA.
23. Reconstitute the Quantibrite PE beads with 250ul PBS+0.09% of NaN3+0.5% of BSA, vortex and add 200ul to the plate
24. Acquire the cells after staining or (if not immediately acquired) store at darkness for max 1 h until measured in the flow cytometer
25. Acquire tube on HTS using setting (table 2 below)
26. Block lid sensor with magnet, pipette up & down 200ul with multichannel pipette each row just before it is acquired by HTS.

**Solutions:**

All the solutions are made with sterile bidestilled water using sterile material to avoid the presence of endotoxin. The solutions have to be filtered with 0,2 µm filters.

- 4% dextran solution (in 0.9% NaCl): 0,9g NaCl, 4g dextran in 100 mL bidestilled water.
- 0.2% NaCl solution: 0,2g NaCl in 100 mL bidestilled water.
- 1.2% NaCl solution: 1,2g NaCl in 100 mL bidestilled water.
- PBS without Ca and Mg plus 2 mM EDTA.
- PBS+0.09% of NaN3+0.5% BSA

Note: Do not decant the tubes. Always use a pipette to discard the supernatants.

**Backbone Table 1**

| Tissue | Target | tube | BV421 | BV510 | FITC | PE | PerCP-Cy5.5 | PE-Cy7 | APC | APC-H7 |
|---|---|---|---|---|---|---|---|---|---|---|
| Blood | Innate cells | 1 | **CD3/19/34/Fixable Violet** | **CD16** | **CD56** | **Test Ab** | **CD14** | **CD123** | **CD11c** | **HLA-DR** |
| | | | | 3G8 | B159 | | M5E2 | 6H6 | B-ly6 | L243 |
| | | | BD | BioLegend | BD | | BD | BioLegend | BD | BD |
| *per test (ul)* | | | 0,50 0,13 0,50 0,50 | 0,38 | 1,25 | | 2,50 | 0,63 | 1,25 | 2,50 |
| ***total No of tests*** | **96** | (ul) | 50,4 12,6 50,4 50,4 | 37,8 | 126 | | 252 | 63 | 126 | 252 |
| | B-T | 2 | **CD27** | **CD45RA** | CD4 **IgD** | **Test Ab** | CD8 **IgM** | **CD19** **TCRgd** | **CD3** | **CD45** |
| | | | O323 | HI100 | MEM-241 IA6-2 | | MEM-31 MHM-88 | LT-19 11F2 | UCHT-1 | MEM-28 |
| | | | BioLegend | BD | Exbio Biolegend | | Exbio BioLegend | Exbio BD | Exbio | Exbio |
| *per test (ul)* | | | 1,00 | 0,80 | 1,90 0,90 | | 0,40 0,90 | 1,10 0,90 | 1,90 | 0,80 |
| ***total No of tests*** | **96** | (ul) | 100,8 | 80,64 | 191,52 | | 40,32 90,72 | 110,9 90,72 | 191,52 | 80,64 |
| tonsil | B cells | 4 | **CD27** | **IgM** | **IgD** | **Test Ab** | **CD3** | **CD19** | **CD138** | **CD38** |
| | | | O323 | MHM-88 | IA6-2 | | SK7 | SJ25C1 | MI15 | HB7 |
| | | | BioLegend | BioLegend | Biolegend | | BD | BD | BD | BD |
| *per test (ul)* | | | 1,00 | 0,75 | 0,63 | | 2,50 | 2,50 | 3,00 | 3,00 |
| ***total No of tests*** | **96** | (ul) | 100,8 | 75,6 | 63 | | 252 | 252 | 302,4 | 302,4 |
| Thymus | Thymocytes | 5 | **CD13/19/33/16/56/Fixable Violet** | **CD4** | **CD44** | **Test Ab** | **CD3** | **CD34** | **CD1a** | **CD8** |
| | | | | OKT4 | G44-26 | | SK7 | 8G12 | HI149 | SK1 |
| | | | BD | BioLegend | BD | | BD | BD | BD | BD |
| *per test (ul)* | | | 0,13 0,13 0,13 0,13 0,13 0,50 | 0,75 | 5,00 | | 2,50 | 2,50 | 2,50 | 0,70 |
| ***total No of tests*** | **96** | (ul) | 12,6 12,6 12,6 12,6 12,6 50,4 | 75,6 | 504 | | 252 | 252 | 252 | 70,56 |

Table 2

| HTS setup | |
|---|---|
| **sample flow rate (u** | **3** |
| **sample volume (uL)** | **150** |
| **mixing volume (uL)** | **100** |
| **mixing speed (uL/se** | **200** |
| **number of mixes** | **2** |
| **wash volume (uL)** | **200** |

Annotation of samples in Diva:

| Annotation field | Keyword | Example | | | |
|---|---|---|---|---|---|
| Well name | CD marker | CD8 | CD28 | CD14 | |
| Patient ID | panel name | 1_DC_mo_Inn | 2_B_T | 4_B | 5_thy |
| Label: | reagent name_fluorochrome | CD4_IgD_FITC | CD19_PC7 | CD28_PE | |
| Sample ID | tissue type | BC01 =buffy coat | | TON01=tonsil | THY01 =thymus |
| Specimen name: | panel name_center_tissue type | 2_B_T_PRG_BC03 | | | |
