# Supplementary material for: CD Maps—Dynamic Profiling of CD1–CD100 Surface Expression on Human Leukocyte and Lymphocyte Subsets
Source: Front Immunol. 2019 Oct 23;10:2434. doi: 10.3389/fimmu.2019.02434 (PMC6820661; doi:10.3389/fimmu.2019.02434)
Supplement: Supplementary file 17 [file Data_Sheet_2.docx]

**Suppl Figure 6.** **Expression levels of all CD markers per cell subset.** Each cell subset is plotted separately with CD marker expression depicted as box-whisker plots depicting antibody binding capacities (ABC). For each subset, the CD markers are ordered from lowest to highest median frequency (black horizontal lines). FMO controls are highlighted and horizontal lines for each FMO sample (dotted lines) and their median (solid line) are shown. Vertical lines show predicted point of change dissecting background levels of expression (to the left of the line) from expressed CD markers (to the right of the line).

**Suppl Figure 7. Individual plots for each cell subset depicting for each CD marker the frequency of positive cells.** For each subset, the CD markers are ordered from lowest to highest median frequency (black horizontal lines). The boxes represent the interquartile ranges and their shades represent the Median antibody binding capacity (ABC) of the marker on that subset. Section highlighted in orange is based on tangent to inflex point in sigmoidal fit.

**Supplementary Figure 8.** Extended version of HCA from main Figure 4.
